# The effect of a movement-to-music video program on the objectively measured sedentary time and physical activity of preschool-aged children and their mothers: A randomized controlled trial

**DOI:** 10.1371/journal.pone.0183317

**Published:** 2017-08-31

**Authors:** Pipsa P. A. Tuominen, Pauliina Husu, Jani Raitanen, Urho M. Kujala, Riitta M. Luoto

**Affiliations:** 1 The UKK Institute for Health Promotion Research, Tampere, Finland; 2 Faculty of Sport and Health Sciences, University of Jyväskylä, Jyväskylä, Finland; 3 Faculty of Social Sciences, Health Sciences, University of Tampere, Tampere, Finland; Universidad de las Palmas de Gran Canaria, SPAIN

## Abstract

Regular physical activity (PA) and the avoidance of prolonged sitting are essential for children’s healthy growth, and for the physical and mental wellbeing of both children and adults. In the context of exercise, music may promote behavioral change through increased exercise adherence and participation. The purpose of this study was to determine whether a movement-to-music video program could reduce sedentary behavior (SB) and increase PA in mother-child pairs in the home environment. A randomized controlled trial was conducted in the Pirkanmaa region, Finland, in 2014–2016. The participants consisted of 228 mother-child pairs (child age 5–7 years). The primary outcomes of interest were tri-axial accelerometer-derived SB and PA, which were measured in weeks one (baseline), two, and eight in both the intervention and control groups. Further, the mothers and children in the intervention group used a movement-to-music video program from the beginning of week two to the end of week eight. Secondary outcomes included self-reported screen time. The statistical methods employed comprised an intention-to-treat and linear mixed effects model design. No statistically significant differences between groups were found in primary or secondary outcomes. Among the children in the control group, light PA decreased significantly over time and screen time increased from 89 (standard deviation, SD 37) to 99 (SD 41) min/d. Among mothers and children in the intervention group, no statistical differences were found. In supplementary analysis, the children who stayed at home instead of attending daycare/preschool had on average 25 (95% confidence interval, CI 19–30) min/d more sedentary time and 11 (95% CI 8–14) min/d less moderate-to-vigorous PA than those who were at daycare/preschool. The higher body mass index of mothers was related with 5 (95% CI 2–7) min/d more sedentary time and 1 (95% CI 0–2) min/d less moderate-to-vigorous PA. The movement-to-music video program did not change the objectively measured SB or PA of the mother-child pairs. However, mothers and children seemed to be more sedentary at home, and therefore interventions for decreasing SB and increasing PA should be targeted in the home environment.

## Introduction

Low levels of physical activity (PA) and high amounts of sedentary behavior (SB), especially excessive sitting, are associated with a higher risk of cardio-metabolic health indicators, obesity, elevated blood pressure, poor physical fitness, and lower academic achievement among both children and adults [1–4]. Regular moderate-to-vigorous PA (MVPA) mitigates these risks and is essential for children’s healthy growth and development, as well as the physical and mental wellbeing of both children and adults [4,5].

The benefits of music in the sports and exercise context have been studied mostly in adults. The effects of music on PA and motivation have been studied, for example, in individual exercise and workouts, such as warm-up [6] and cool-down routines [7,8], strength-based workouts [9], cardio-respiratory workouts, especially running [10,11] and cycling [12,13], and classes and group activities with music, specifically aerobics [14] and circuit training [15]. It has been found that during exercise, motivational music may enhance the effect, reduce ratings of perceived exertion, improve energy efficiency, and lead to increased work output [8,16,17]. In addition, music could promote behavioral change with increased exercise adherence and participation [18].

Among children, music is often included in PA programs for children with disabilities by using rhythms, instructions set to music, listening to music, and movement-to-music in order to motivate children to engage in PA [19]. The preschool-aged (5–6-years-olds) healthy children’s favorite musical activities in daycare have been found to be movement-to-music (for example, dancing), singing songs, and playing instruments [20]. Further, regularly provided, structured PA programs with music have been found to increase the amount and intensity of PA in children, and to improve their motor skills [21]. We conducted a pilot study (n = 24 mother-child pairs) that found that over two weeks (baseline and intervention), the mothers and children who used a movement-to-music video program (i.e., the intervention group) demonstrated less sedentary time during the intervention week compared to baseline week: the opposite was true in the control group [22]. However, to our knowledge, high-quality randomized controlled trials (RCT) have not been conducted in the area of movement-to-music-based exercise, SB, and PA. Thus, in light of the earlier studies, it is unclear whether the use of music as part of an exercise program can reduce SB and increase PA in the long term.

Studies on the intergenerational transmission of SB and PA have shown that parents play a critical role in their children’s SB and PA [23–25]. Both the PA and TV viewing of parents are significantly associated with these behaviors in preschool children [26], especially during weekends [27]. Rebold et al. (2016) found that parental direct supervision and participation during PA is an important factor in improving children’s PA behavior [28]. Further, Xu et al. (2014) reported that parent’s support for PA can increase their children’s PA, and the parent acting as a role model by watching less TV can lead to the decreased screen time of their children [25]. In addition, the influence of the sex-matched parent appeared to be important for the children’s TV viewing [26]. It has been suggested that the effects of PA and SB interventions may be stronger for children whose parents meet the PA recommendations, who are active and participate in sports, and who have fewer media devices at home [24,29].

The PA guidelines for adults recommend at least 150 min of moderate-intensity (3–6 METs, metabolic equivalent) or 75 minutes of vigorous (≥ 6 METs) PA, or an equivalent combination of aerobic activities every week in sessions of 10 min or more [5]. Muscle-strengthening activities and/or balance training for all major muscle groups are also recommended on two or more days a week [5]. The importance of lifestyle counseling and motivating exercise programs is obvious, because based on objective measurement by accelerometer, recent studies have shown that adults are sedentary 55–62% of their waking hours and MVPA covers only 4–6% of the measurement time [30,31]. In Finland, under a quarter of adults meet the aerobic part of the current PA recommendations, and among women, aged 30–39 years, SB accounted for 57%, standing still (SS) 18%, light PA 16%, and MVPA 10% of the waking wear time per day [31].

In Finland, children start school in the autumn of the year, they turn 7 years of age. One year before school age, children must take part in a year-long preschool organized in day-care centers and schools. Thus, the exact age of preschoolers varies from 5 to 7 years, depending on the birth date of the child and the time of assessment. At the same time, children have a right to attend daycare (part time) or stay at home with a parent or other nursing staff. For children under 7 years, the most recent PA guidelines recommend at least 180 minutes activity at any intensity spread throughout the day [32–36]. According to a review by Hnatiuk et al. (2014), the proportion of time preschool children spent sedentary has been reported to range from 34% to 94%, the proportion of light PA from 4% to 33%, and proportion of MVPA from 2% to 41% [37]. In Finland, the amount of time children spend sedentary is high, and overall PA levels are low [38]. Further, for most of the time spent at childcare, PA levels and activity types are sedentary in nature [39]. Based on accelerometer measurements, the sedentary time was 5.5 hours per day in 3–6-year-old children [40,41]. In addition, Finland’s most recent Report Card on Physical Activity (2016) states that only 29% of three-year-old children and 49% of primary-school-aged children engage in at least 60 minutes of MVPA [41]. There is a gap in objectively measured SB and PA data regarding preschool-aged children. Such information would help to follow changes in SB and PA through childhood and to target activities at those children who need it the most.

Thus, the purpose of the present study was to investigate the effects of a movement-to-music video program on SB and PA in 5–7-year old children and their mothers. We tested the hypothesis that the movement-to-music video program developed for mother-child pairs would i) decrease the SB of mothers and their children, and ii) increase the amount of their PA. The outcomes were objectively measured by accelerometers.

## Materials and methods

The current randomized controlled trial (RCT) was registered at ClinicalTrials.gov (NCT02270138). The study was approved by the Pirkanmaa Ethics Committee in Human Sciences (ETL-Code R14039, statement 23/2014), and all mothers gave informed consent on their own and their child’s behalf. The study was conducted in accordance with prevailing ethics principles. The reporting of the methods and findings of this trial was guided by the CONSORT 2010 checklist for reporting randomized trials [42].

### Participants

Participants were mothers and their children recruited between November 2014 and January 2016 from the cohort of NELLI: Pregnancy as a window to the future health of mothers and children: the 7-year follow-up of a gestational lifestyle intervention in the Pirkanmaa area, Finland (ISRCTN33885819; see http://www.controlled-trials.com/). The rationale and methods of the current study have been published previously by Tuominen et al. (2015) [43]. The following inclusion criteria were used: child included in the original NELLI cohort, child aged 5–7 years, family had access to a DVD player or could watch a YouTube video, both mother and child could use the accelerometer as instructed, and neither the mother nor the child had any obstacles to performing PA.

Study information was given to the mothers both orally and in writing during the contact for the examination that was part of the NELLI study. If the mother was willing and the mother and child eligible to participate in the study, the mother-child pair was randomized into either the intervention group or the control group by means of sealed envelopes by laboratory staff. Randomization was performed with a random number generator for blocks of four mother-child pairs in a 2:2 ratio: two mother-child pairs were assigned to the intervention group and two pairs to the control group. Four random numbers were generated, and the pairs associated with the two largest were assigned to the intervention group and the two lowest to the control group. After randomization neither the participants nor the researchers were blinded.

### Intervention

All mothers and children were instructed to use an accelerometer every day during waking hours for weeks one (baseline), two, and eight. In addition, all mothers completed exercise diaries for themselves and their child for the same weeks. The first measurement week (i.e., week one) was used as the baseline measurement in both groups before the start of the intervention. Further, the mothers and children in the intervention group were instructed to use the movement-to-music video program DVD every other day from the beginning of week two to the end of week eight. The movement-to-music video program consisted of three separate exercise programs, each lasting 10 minutes. As per the instructions, the videos could be used individually or consecutively in order to allow the mother and child to choose the suitable amount of exercise for themselves. The contents of video program have been previously described by Tuominen et al. (2015) [43].

Mothers and children who used accelerometers for at least four days during any of the measurement weeks and for at least 10 hours per day were included in the analysis. Participants whose daily measurement time exceeded 20 hours were considered to have slept with the accelerometer. Thus, to avoid possible bias in SB time, their waking wear time was limited to 20 hours, with the deduction coming from their lying-down time (or from sitting time, if the lying-down time was shorter than the exceeded proportion). The variables of SB and PA are presented as a proportion of the total measurement time (waking hours) during measurement days.

### Measurements

The primary outcomes of the study were SB and PA, which were assessed objectively by means of the accelerometer (Hookie AM20, Traxmeet Ltd, Espoo, Finland), and further examined via the exercise diaries and questionnaires. The accelerometer collected and stored the tri-axial acceleration signal in raw mode with a 100 Hz sampling frequency and a ±16 g (the Earth’s gravity) measurement range caused by any movement. The collected raw acceleration data were transformed into actual g-units [44,45]. The data was analyzed as the mean signal amplitude deviation (MAD) of the resultant acceleration for each epoch [44]. The resultant, which indicates the magnitude of the acceleration, was calculated for every measured sample. The data were analyzed at a 6-second epoch length.

Standing, sitting, and lying down were identified by applying the tri-axial information from the accelerometer. Walking was used as a reference and since the body posture during walking is upright and the direction of Earth’s gravity vector is constant, the vertical position (angle) of the accelerometer can be identified during normal walking. This known position (i.e., the angle of the accelerometer) can then be used for the purposes of recognizing different body postures. In standardized conditions, standing can be separated from sitting or lying with 100% accuracy, sitting from lying with 99% accuracy, and standing from sitting with 93% accuracy [31,46]. Lying and sitting down (<1.5 MET) was combined as SB, while standing still (SS < 1.5 MET) and light PA (LPA 1.5–2.9 MET) were analyzed separately [47,48]. Moderate-to-vigorous PA (MVPA) consisted of moderate PA (MPA 3.0–5.9 MET) and vigorous PA (VPA ≥6.0 MET) [48]. The accelerometer has been shown to be a valid measurement tool among adults [45] and young people [49,50]. Based on our pilot study, the Hookie-accelerometer is a feasible tool also among preschool children [22].

For the exercise diary data, mothers were asked to record their working hours and the start and end times of PA (such as walking, jogging, running, swimming, biking, gym workouts, and dancing). In addition, the mothers were given diaries for the children, in which they recorded the child’s daycare or preschool time, exercises, and the time spent engaged in PA.

The secondary outcome of the study was screen time, which was evaluated by means of self-reported information at baseline and after eight weeks via a questionnaire. The baseline questionnaire also included information on the participants’ background, socioeconomic status, PA, screen time, height, weight, musculoskeletal disorders or symptoms, and perceived health status. The questionnaire on the participants’ current PA and time spent in a sitting position was utilized in the national Health 2011 Survey [51] and the FINRISKI study [52] in Finland to ascertain whether or not people meet the PA recommendations and how much they tend to sit in various contexts (in the office, at home in front of the computer or TV, during transportation). Disorders and symptoms, as well as perceived health were considered important elements in functional capacity and health [53]. Perceived health was measured via a visual analog scale (VAS). The questionnaire for the children was based on the LATE (Health monitoring among children and youth in Finland) project [54]. Parents reported their child’s behavior, and the questionnaire included separate questions on outside activities, exercises, and screen time.

### Statistical analysis

Sample size calculation was based on the Moving Sound pilot study [22], where the mean sedentary time of the mothers was 7 h 40 min per day at baseline. The average reduction in sedentary time in the intervention group at the end of the study was assumed to be around 6%, while the control group would remain unchanged. Power calculations for the study have been reported earlier by Tuominen et al. (2015). Briefly, differences in groupwise means were tested via *t*-tests. When the two-sided significance level was 0.05 and the power of the study was 80%, the effect size varied from 0.357 to 0.500 (depending on the changes in sedentary time). Based on these calculations, the estimated sample size for the study was 63–124 mother-child pairs per group [43].

Baseline characteristics and primary and secondary outcomes were reported as means and standard deviations (SD) for continuous variables and as frequencies and percentages for categorical variables, since the data were normally distributed based on values of skewness and kurtosis. The primary outcomes (proportion of measurement time in SB, SS, LPA, and MVPA) were analyzed on the basis of the linear mixed effects model (LME) with group (intervention or control), time (day), and interaction between group and time. Unstructured covariance type was used for repeated measurement analysis, based on the assumption that every term (the variances and the correlation between two separate measurements) may be different [55]. Further, the model for mothers was adjusted for the mother’s body mass index (BMI), number of children, working status (yes/no), self-reported musculoskeletal disorders or symptoms (yes/no), and perceived health status (VAS 0–100). The model for children was adjusted for the child’s BMI, daycare or preschool (yes/no), and number of siblings. Potential confounding factors were included in the analyses by adding them one by one to the model as far as the estimate for interaction term changed in the primary outcomes. The change of the estimate for interaction term was not essential when the child’s age or gender was added to the model, and therefore they were omitted. The self-reported secondary outcome (screen time) was analyzed using a LME model within two time points (baseline and end). Outliers were removed prior to the analysis if standardized values (z-score) were less than -3.30 or greater than 3.30. A Pearson correlation coefficient was computed to assess the relationship between the mothers’ and children’s SB, PA, and screen time.

All the data were analyzed using the intention-to-treat analysis principle. A mixed effects model uses all data available at each time point (i.e., every measurement day); thus, data from accelerometers during weeks 1, 2, and/or 8 were used when the mothers and/or children had the data for at least four days per week. As sensitivity analyses, we also performed the LME models for the mothers and children who responded to all three time points (i.e., had acceptable measurements from weeks one, two, and eight), and, also for those mothers and children who used the movement-to-music video program (based on the diaries) during week 8 (the last intervention week).

In the dropout analysis, Fisher’s exact test was used for dichotomous variables (group, gender of the child, being in work, the child staying in daycare or preschool), and the independent samples *t*-test was used for continuous variables (age, BMI, perceived health) to find out whether there were differences between those who discontinued the study compared to those who continued until the end. All analyses were performed with IBM SPSS Statistics 24.0.

## Results

**Fig 1:**
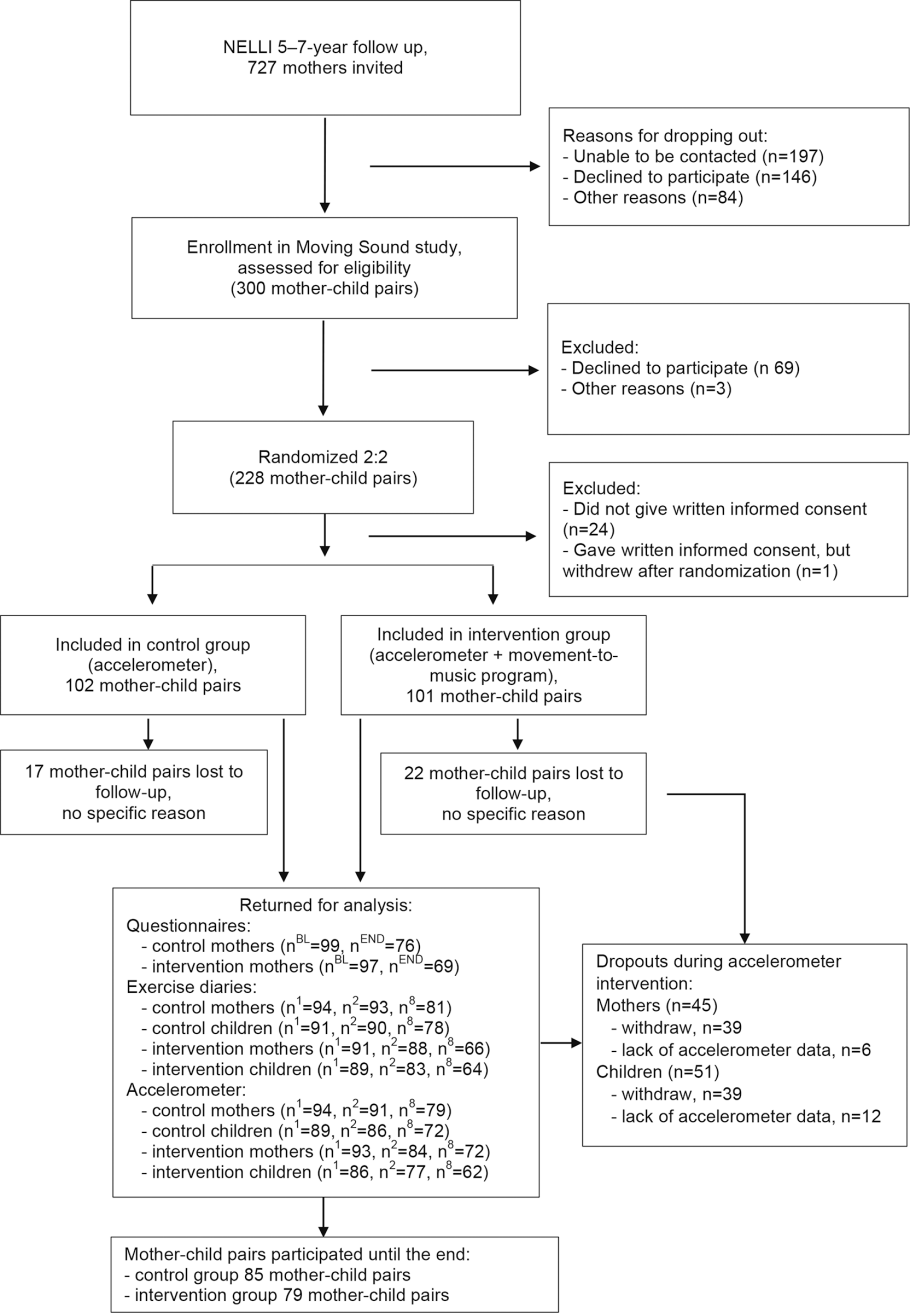
Flowchart of the study. (Abbreviations: n = number of participants, n^BL^ = number of participants at baseline, n^END^ = number of participants at the end, n^1^ = number of measured participants at the first week, n^2^ = number of measured participants at the second week, n^8^ = number of measured participants at the eighth week).

There were 228 mother-child -pairs who were randomized (Fig 1), but 13 mothers in the intervention group and 11 mothers in the control group withdrew immediately after randomization or did not return the signed informed consent, and were therefore excluded from the data. One mother, who was randomized to the control group, returned an informed consent signed, but did not want to wear an accelerometer and did not return any other data. She was therefore also excluded. In total, 203 mother-child pairs were included (intervention group 101 mother-child pairs, control group 102 mother-child pairs) in the intention-to-treat analysis. Of the mother-child pairs included, 164 (81% of included participants) completed the study up to the 8-week time (intervention group 79 mother-child pairs, control group 85 mother-child pairs). A mother-child pair was considered to participate until the end of the study if the mother returned the accelerometer, exercise diary, and/or questionnaire after the whole intervention period.

The background characteristics of the participants are presented in Table 1. The mothers in the intervention and control groups did not differ from each other. Regarding the children, the intervention group seemed to include more boys than girls, while the control group had slightly more girls than boys. However, the differences between the groups were not statistically significant. In addition, based on the Consort 2010 statement, any differences in baseline characteristics are the result of chance rather than bias [56] (http://www.consort-statement.org/checklists/view/32-consort/510-baseline-data).

**Table 1:** Background characteristics of the participants. (Abbreviations: n = number of participants, SD = standard deviation).

	Intervention		Control	
	n	Mean (SD) / %	n	Mean (SD) / %
**Mothers**				
Age (in 2015)	101	37.0 (4.7)	102	37.9 (5.0)
Marital status	97		99	
married		77.3%		78.8%
cohabiting		13.4%		19.2%
divorced		6.2%		2.0%
unmarried		3.1%		-
Employment	97		99	
full- or part-time work		69.1%		76.8%
maternity, parental, or child care leave		13.4%		11.1%
unemployed or laid off		7.2%		5.1%
other		10.3%		7.1%
Pregnant	97		99	
no		96.9%		98.0%
yes		3.1%		2.0%
BMI (includes only non-pregnant women with measured weight)	94	27.7 (5.3)	93	26.2 (4.7)
Musculoskeletal disorders	96		96	
no		90.6%		88.5%
yes		9.4%		11.5%
Musculoskeletal symptoms	94		96	
no		23.4%		30.2%
yes		76.6%		69.8%
Perceived health	96	74.5 (13.4)	99	76.3 (12.1)
**Children**				
Age (at the beginning of the measurements)	101	6.5 (0.5)	102	6.5 (0.5)
Gender	101		102	
girl		44.6%		54.9%
boy		55.4%		45.1%
BMI (based on measured weight, transmitted to adult scale)	99	21.9 (4.4)	97	21.8 (4.0)
girl	44	20.7 (3.3)	50	20.9 (3.7)
boy	55	22.8 (4.9)	47	22.8 (4.1)
Childcare or preschool at least three days per week	101	65.3%	102	65.7%

Using a visual analog scale (VAS), the mean of the mothers’ self-reported perceived health was 74.5 (SD 13.3) in the intervention group and 76.3 (SD 12.1) in the control group, indicating a fairly good health status in both groups. Based on the mothers’ self-reports, 33% of the intervention group (n = 97) and 39% of the control group (n = 97) met the PA recommendation for aerobic PA, and 30% of the intervention group and 32% of the control group met the muscle-strengthening/balance recommendation. However, only 12% of the intervention group and 14% of the control group mothers met both recommendations.

Accelerometer data (at least four days per week and 10–20 hours/day) was used in the analysis if there was at least one acceptable measurement week for the accelerometer data (see Table 2).

**Table 2:** The use of the accelerometer over the study: number of users (n), weekly average (days/week), and daily average (SD) in hours (h).

	Week 1 (baseline)	Week 2 (the first intervention week)	Week 8 (the last intervention week)	Acceptable measurement data for any week	Acceptable measurement data for all weeks
**Mothers**					
Control group (n = 102)	n = 94, 6.73 d/wk, 14.7 h (1.3)	n = 91, 6.26 d/wk, 14.5 h (1.1)	n = 74, 6.60 d/wk, 14.6 h (2.0)	94%	68%
Intervention group (n = 101)	n = 93, 6.67 d/wk, 14.6 h (1.1)	n = 84, 6.24 d/wk, 14.5 h (1.2)	n = 65, 6.61 d/wk, 14.6 h (1.9)	95%	60%
**Children**					
Control group (n = 102)	n = 89, 6.43 d/wk, 13.2 h (1.2)	n = 86, 6.05 d/wk, 13.1 (1.3)	n = 63, 6.17 d/wk, 13.1 h (2.0)	89%	60%
Intervention group (n = 101)	n = 86, 6.52 d/wk, 13.3 h (1.3)	n = 77, 5.99 d/wk, 13.4 h (1.2)	n = 56, 6.56 d/wk, 13.5 h (2.1)	87%	50%

### Mothers

**Fig 2:**
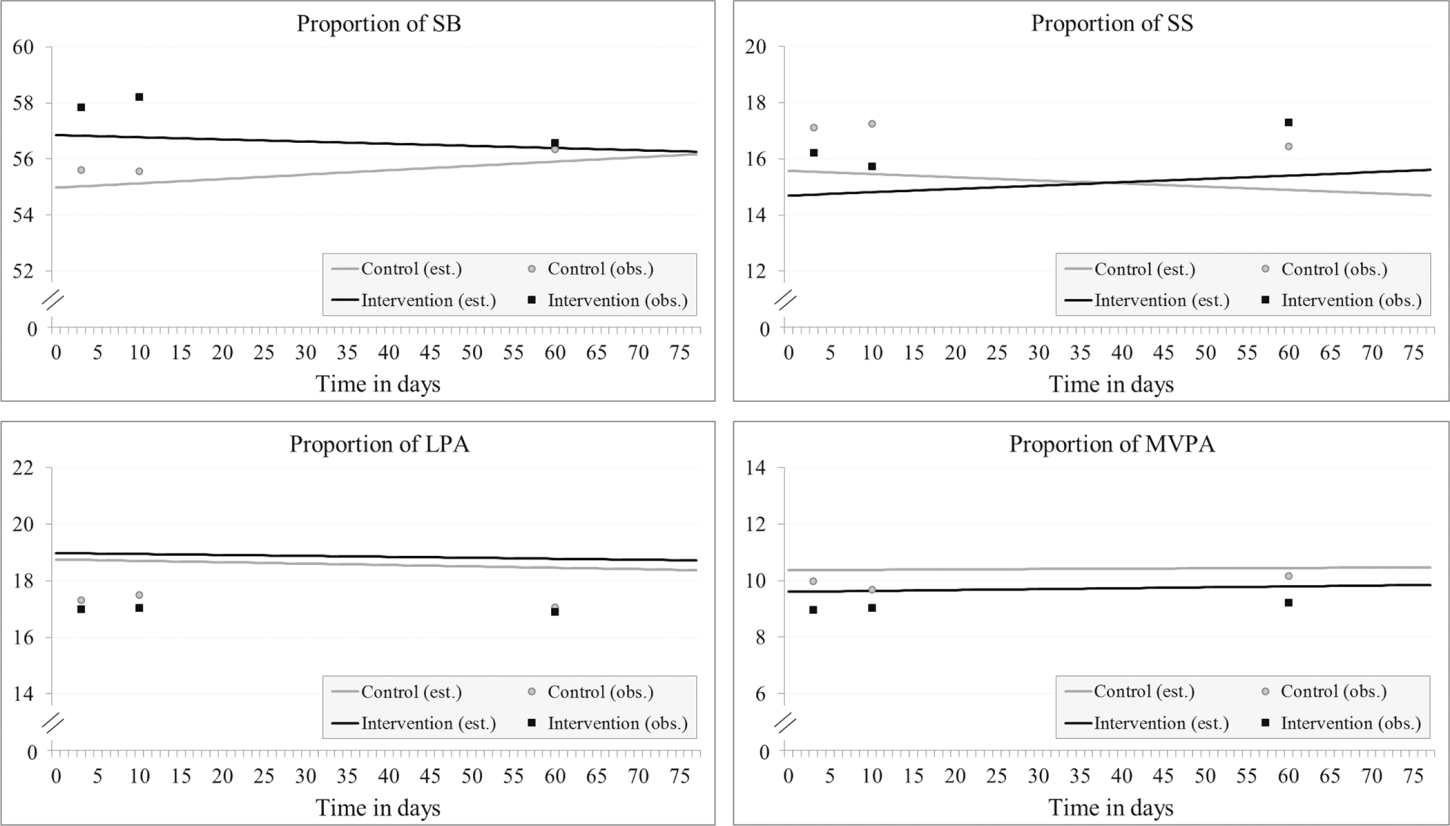
The proportion of sedentary behavior (SB), standing still (SS), light physical activity (LPA), and moderate-to-vigorous physical activity (MVPA) per day as a weekly average and a trend of change over time among mothers. (Abbreviations: est. = estimated, obs. = observed).

At baseline, the proportion of sedentary time was higher in the intervention group compared to the control group, but the difference was not statistically significant. Over the study period, the proportion of SB decreased slightly in the intervention group and increased in the control group, but the change in time between groups was not significant, nor were the changes over time within groups. The adjusted model did not show a significant difference over time between the groups either (Table 3, Fig 2).

**Table 3:** Change within and between the groups of mothers in sedentary behavior and physical activity over time as a proportion of measurement time (estimates, 95% confidence intervals and p-value).

		Unadjusted (n = 191)		Adjusted* (n = 170)	
Mothers		estimate (95% CI)	*p*-value	estimate (95% CI)	*p*-value
Sedentary behavior**					
	difference at baseline (ref = control)	1.89 (-0.6 to 4.4)	0.135	1.87 (-0.61 to 4.36)	0.139
	change in time, control	0.008 (-0.015 to 0.03)	0.486	0.015 (-0.010 to 0.041)	0.234
	change in time, intervention	-0.009 (-0.034 to 0.017)	0.495	-0.008 (-0.034 to 0.019)	0.566
	intervention effect (ref = control)	-0.017 (-0.052 to 0.015)	0.330	-0.023 (-0.060 to 0.014)	0.215
Standing still**					
	difference at baseline (ref = control)	-1.01 (-2.38 to 0.37)	0.150	-0.88 (-2.33 to 0.56)	0.228
	change in time, control	-0.008 (-0.024 to 0.008)	0.329	-0.011 (-0.028 to 0.006)	0.191
	change in time, intervention	0.014 (-0.003 to 0.031)	0.101	0.012 (-0.006 to 0.029)	0.183
	intervention effect (ref = control)	0.022 (-0.001 to 0.045)	0.063	0.023 (0.001 to 0.048)	0.063
Light physical activity**					
	difference at baseline (ref = control)	-0.067 (-1.29 to 1.16)	0.914	-0.22 (-1.51 to 1.06)	0.730
	change in time, control	-0.003 (-0.015 to 0.008)	0.580	-0.005 (-0.017 to 0.007)	0.433
	change in time, intervention	-0.003 (-0.015 to 0.009)	0.621	-0.003 (-0.016 to 0.009)	0.606
	intervention effect (ref = control)	0.0002 (-0.016 to 0.017)	0.983	0.002 (-0.016 to 0.019)	0.860
Moderate-to-vigorous physical activity**					
	difference at baseline (ref = control)	-0.82 (-1.72 to 0.08)	0.073	-0.77 (-1.71 to 0.17)	0.109
	change in time, control	0.003 (-0.006 to 0.011)	0.489	0.001 (-0.008 to 0.011)	0.786
	change in time, intervention	-0.0003 (-0.009 to 0.009)	0.951	-0.001 (-0.010 to 0.009)	0.909
	intervention effect (ref = control)	-0.003 (-0.016 to 0.009)	0.603	-0.002 (-0.015 to 0.012)	0.786

* Adjusted for mother's BMI, number of children, work, disorders or symptoms, and perceived health

** Proportion of measurement time

The proportion of SS at baseline was lower in the intervention group than in the control group, but the difference was not significant. Over time, the proportion of SS slightly increased in the intervention group and decreased in the control group, but the change over time between the groups was not significant in either the unadjusted or adjusted (*p* = 0.063, separately) model.

The proportion of LPA was higher and MVPA was lower at baseline in the intervention group than in the control group, but the differences were not significant. In both the unadjusted and adjusted model, changes in LPA and MVPA were very small in both groups, and there were no differences within or between the groups in either the unadjusted or adjusted model.

Although all the changes in primary outcomes were non-significant, we found that at the baseline each additional unit of BMI added 0.5%-point the proportion of SB, when controlling for covariates (*p*<0.001). The adjusted model also showed that each additional unit of BMI lowered the proportion of SS (*p* = 0.007), LPA (*p* = 0.004), and MVPA (*p* = 0.008) by 0.13–0.19%-point. When the results are expressed in terms of minutes per day, these numbers varied between one and five minutes per day when controlling for waking wear time and other covariates. Further, if the mother was not working outside the home, her proportion of SS was 2.3%-point lower (*p* = 0.013) compared to working mothers, which means around 18 min less standing still per day.

Based on the self-reported data (n = 192) mothers in the intervention group had on average screen time of 124 (SD 54) min/d at baseline and the mothers in the control group had on average screen time of 132 (SD 62) min/d. Over time, screen time increased in both groups by two to three minutes per day, and none of the changes within or between groups was statistically significant. However, the proportion of mothers in the intervention group having a screen time of more than two hours per day increased over the study (baseline 51%, end 62%), while in the control group the proportion decreased (baseline 57%, end 53%).

Based on the questionnaire data, 57 mothers in the intervention group reported at least one session of exercise with the movement-to-music video program during week 2 (the first intervention week). Mothers performed on average 2.7 exercise sessions (SD 1.0) lasting on average 19 min/session (SD 8.0). After the intervention, the mothers were asked to evaluate how many times per week and how long they had exercised during the intervention period. On average, 1.7 exercise sessions (SD 1.0) lasting 17 min (SD 9.1) were reported by 33 mothers.

### Children

**Fig 3:**
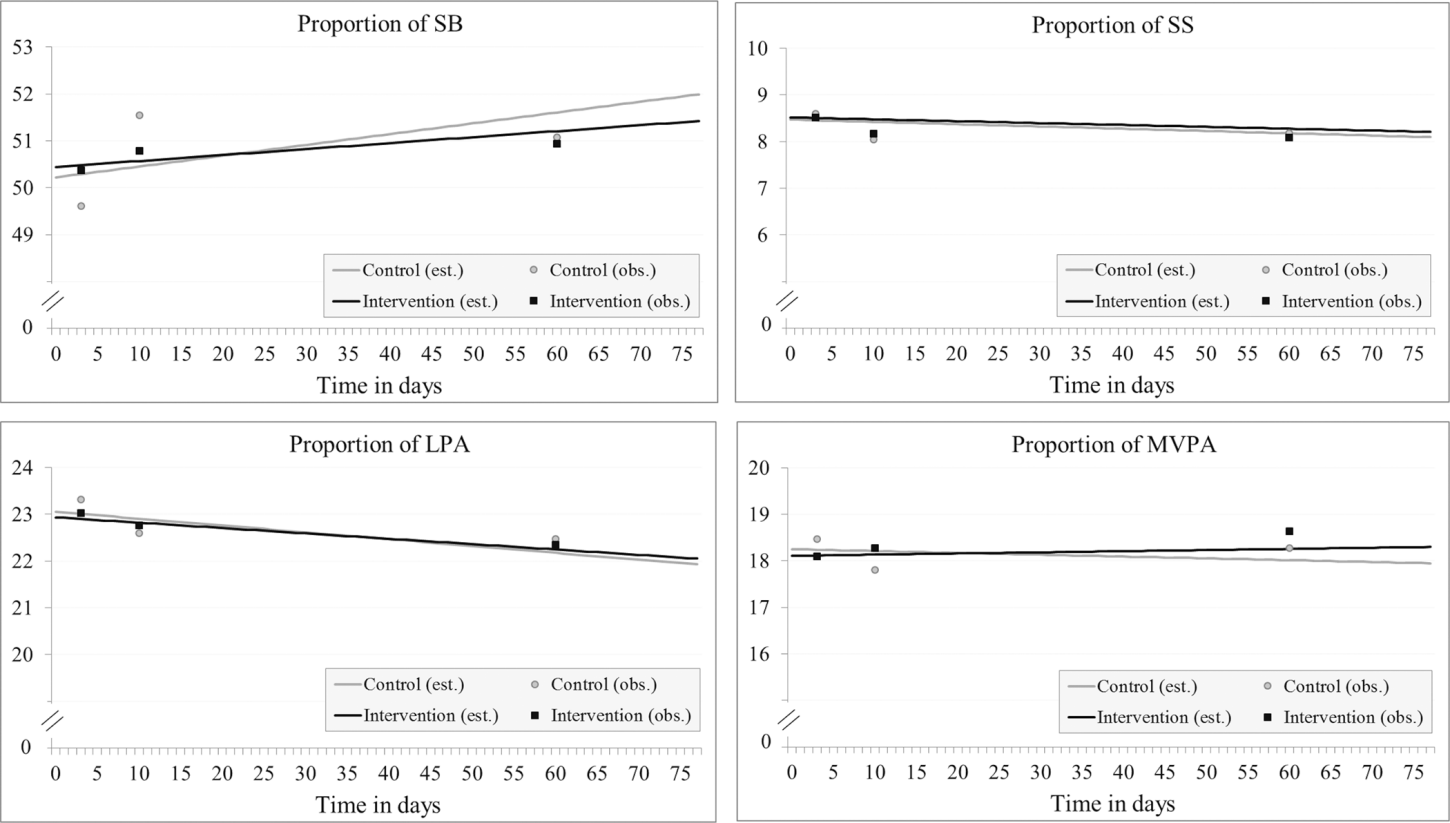
The proportion of sedentary behavior (SB), standing still (SS), light physical activity (LPA), and moderate-to-vigorous physical activity (MVPA) per day as a weekly average and a trend of change over time among children. (Abbreviations: est. = estimated, obs. = observed).

The proportion of SB at baseline was higher in the intervention group than in the control group in both the unadjusted and adjusted models, but the difference was not significant in either model. Over time, the proportion of SB tended to increase in both groups, but the change in time between groups was not significant (Table 4, Fig 3).

**Table 4:** Change within and between the groups of children in sedentary behavior and physical activity over time as a proportion of measurement time (estimates, 95% confidence intervals and p-value).

		Unadjusted (n = 180)		Adjusted* (n = 170)	
Children		estimate (95% CI)	p-value	estimate (95% CI)	p-value
Sedentary behavior**					
	difference at baseline (ref = control)	0.017 (-1.91 to 1.94)	0.986	0.219 (-1.69 to 2.28)	0.825
	change in time, control	0.019 (-0.006 to 0.045)	0.134	0.023 (-0.003 to 0.049)	0.085
	change in time, intervention	0.012 (-0.014 to 0.039)	0.360	0.013 (-0.013 to 0.039)	0.335
	intervention effect (ref = control)	-0.007 (-0.044 to 0.029)	0.697	-0.010 (-0.047 to 0.027)	0.583
Standing still**					
	difference at baseline (ref = control)	0.209 (-0.508 to 0.925)	0.566	0.046 (-0.700 to 0.792)	0.904
	change in time, control	-0.003 (-0.013 to 0.007)	0.584	-0.005 (-0.015 to 0.005)	0.347
	change in time, intervention	-0.004 (-0.014 to 0.006)	0.437	-0.004 (-0.014 to 0.006)	0.437
	intervention effect (ref = control)	-0.001 (-0.016 to 0.013)	0.857	0.001 (-0.014 to 0.015)	0.907
Light physical activity**					
	difference at baseline (ref = control)	-0.090 (-1.084 to 0.903)	0.858	-0.117 (-1.138 to 0.904)	0.821
	change in time, control	-0.015 (-0.026 to -0.003)	**0.015**	-0.015 (-0.027 to -0.002)	**0.019**
	change in time, intervention	-0.011 (-0.023 to 0.001)	0.065	-0.011 (-0.024 to 0.001)	0.064
	intervention effect (ref = control)	0.003 (-0.014 to 0.020)	0.712	0.003 (-0.014 to 0.020)	0.717
Moderate-to-vigorous physical activity**					
	difference at baseline (ref = control)	-0.128 (-1.289 to 1.034)	0.828	-0.140 (-1.315 to 1.035)	0.814
	change in time, control	-0.003 (-0.018 to 0.012)	0.711	-0.004 (-0.019 to 0.012)	0.619
	change in time, intervention	0.003 (-0.013 to 0.018)	0.729	0.002 (-0.013 to 0.018)	0.753
	intervention effect (ref = control)	0.005 (-0.016 to 0.027)	0.613	0.006 (-0.016 to 0.028)	0.565

* Adjusted for child's BMI, daycare or preschool, and number of siblings

** Proportion of measurement time

The proportion of SS at baseline was on average higher in the intervention group than in the control group in both the unadjusted and adjusted model, but the difference was not significant. Over time, the proportion of SS tended to decrease in both groups, but the change in time between the groups was not significant.

The proportions of LPA and MVPA at baseline were on average lower in the intervention group than in the control group in both models, but the differences between groups were not significant. Over time, the proportion of LPA decreased both in the intervention group (unadj. *p* = 0.065, adj. *p* = 0.064) and in the control group (unadj. *p* = 0.015, adj. *p* = 0.019). Over time, the proportion of MVPA tended to increase in the intervention group and decrease in the control group, but the changes within groups were not significant. Although the changes over time in MVPA were in opposite directions, the difference between groups in terms of LPA or MVPA was not significant either.

As with the mothers, all the changes in primary outcomes were small. However, we found that if the child was at home instead of daycare or preschool, at the baseline he/she had a 1.9%-point higher proportion of SB, a 0.8%-point lower proportion of SS, and a 0.8%-point lower proportion of MVPA compared to those who were in daycare or preschool in the adjusted model (*p*<0.001, separately). Children who were at home had a 0.3%-point lower proportion of LPA, but the difference for those who were in daycare or preschool did not reach the level of statistical significance (*p* = 0.080). When the results are expressed in terms of minutes per day, children who were at home had 25 min (95% CI 19–30) more sedentary time per day compared to those who were in daycare or preschool when controlling for waking wear time and other covariates. The amount of SS was 8 min (95% CI 6–10), LPA 6 min (95% CI 4–8), and MVPA 11 min (95% CI 8–14) lower among children who were at home compared to children in daycare or preschool. We also found that each additional unit of BMI decreased the proportion of LPA by 0.2%-point (*p* = 0.007, 1.2 min/d).

Based on self-reported data (n = 193), screen time among children in the intervention group was on average 82 min per day (SD 37) at baseline and 89 min/d (SD 37) in the control group, but the difference between the groups at baseline was not statistically significant. Over time, screen time increased on average to 83 min/d (SD 37) in the intervention group and to 99 min/d (SD 41) in the control group. The difference between the baseline and end was significant in the control group (*p* = 0.031), but not in the intervention group or between the groups. Further, the proportion of children in the intervention group having a screen time more than two hours per day remained at the same level over the study (baseline 20%, end 21%), while in the control group the proportion increased (baseline 21%, end 32%).

Based on the questionnaire data, 61 children in the intervention group were reported to have had at least one exercise session during the intervention. Mothers reported that the children had on average 2.7 exercise sessions (SD 1.1) lasting on average 19 min/session (SD 8.4) with the movement-to-music video program during week 2 (the first intervention week). After the intervention, the mothers were asked to evaluate how many times per week and for how long their children had exercised during the intervention period, and on average 1.9 sessions (SD 1.1) lasting 17 min (SD 8.5) was reported among 36 children.

### Relationship between mothers’ and children’s SB, PA, and screen time

There were either no correlations or weak correlation (r = 0.03–0.20) found between the mothers and children in SB, SS, LPA, and MVPA. The relationships were slightly stronger, but still weak (r = 0.07–0.30), when both the mother and child spent the day at home.

A moderate correlation (r = 0.41) was found between the self-reported screen time of mothers and children. The correlation was slightly stronger in the intervention group (r = 0.43) than in the control group (r = 0.39). In the intervention group, the boys’ screen time correlated with the mothers’ screen time slightly more than the girls’ screen time (r = 0.45 vs. r = 0.40), while the corresponding values in the control group were r = 0.51 (boys) vs. 0.31 (girls).

### Sensitivity analysis

Sensitivity analysis for those mothers who had acceptable accelerometer use for all three weeks (n = 130) did not show any significant differences or changes within or between groups at baseline or over time in the unadjusted or adjusted model (S1 Table).

The corresponding sensitivity analysis for children (n = 111) showed some differences in the unadjusted and adjusted models compared to the intention-to-treat analysis in the proportion of LPA, but not in the other variables (S2 Table). The analysis showed that the difference in the proportion of LPA was not significant at baseline. However, a statistically significant difference within groups occurred over time, with the proportion of LPA decreasing both in the intervention group and in the control group. No significant difference between groups was found over time.

Sensitivity analysis for those mothers who used the movement-to-music video program (based on diaries, n = 9) during week 8 or belonged to the control group (n = 96) did not show any further results (S3 Table).

The corresponding sensitivity analysis for children (intervention group n = 10, control group n = 91) showed similar results as in the intention-to-treat analysis (S4 Table). The difference in the proportion of LPA was not significant at baseline. Over time, a statistically significant difference within the control group occurred, with the proportion of LPA decreasing. Significant differences over time were not found within the intervention group or between the groups.

### Dropout analysis

As mentioned earlier, participants were considered to have participated until the end of the study if they had returned the accelerometer, exercise diary, and/or questionnaire. At least some data for the eighth week were available for 81% of 203 mother-child pairs. During the accelerometer intervention, 22% of the mothers and 25% of the children were lost.

Compared to the mothers for whom the accelerometer data during the eighth week were not available at all (n = 45), mothers with at least some accelerometer data (n = 158) were more likely to work or study outside the home (n = 154, 84% vs. n = 42, 64%, *p* = 0.008). There were no statistical differences between these two groups in belonging to the intervention or control group, age, being married or cohabiting compared to divorced or unmarried, being pregnant or non-pregnant, BMI, having at least one musculoskeletal disorder or symptom, or perceived health (VAS scores).

Corresponding to the mothers, compared with the children for whom the accelerometer data during the eight-week period were not available at all (n = 51), children with at least some accelerometer data (n = 152) were more likely to stay in daycare or preschool for at least three days per week for a minimum of two weeks (78% vs. 29%, *p*<0.001). There were no statistical differences between these two groups in belonging to the intervention or control group, gender, age, or BMI.

## Discussion

The aim of the present study was to investigate the effects of the movement-to-music video program on objectively measured SB, SS, LPA, MVPA of the mothers and their children. The present study also reported the self-reported screen time.

During the baseline week, mothers in the intervention group had on average slightly more SB, less SS, slightly more LPA, and less MVPA than average Finnish women in the Finnish Health 2011 Study [31]. The corresponding results in the control group indicated less SB and SS, slightly more LPA and the same amount of MVPA than Finnish women an average [31]. We did not find any statistically significant differences between mothers in the intervention and control groups either in SB or PA outcomes at baseline or over the study. Thus, our results are converse to previous studies for using music as a tool for less SB or for more PA [16–18].

During the baseline week, children spent an average 50% (6.6 hours per day) of their waking time sedentary in both groups. This is approximately one hour more sedentary time per day than reported in the Finland’s latest Report Card [41], and the situation was even worse during the second and eighth week. However, children spent an average 23% of their waking time in LPA and 18% in MVPA (together, 5.4 hours per day), which is considerably more than the most recent PA guidelines recommend for children [32–36]. In addition, the proportion of MVPA seemed to increase slightly during the second and eighth week among the intervention children, but the change was not statistically significant. Ward et al. (2010) reported that a PA program with music increased the amount and intensity of PA in children [21], which is in line with our study. However, it is possible that small changes over time can be explained by the motivational effect of the accelerometer rather than the effect of the movement-to-music video. Thus, our results differed from the Dance, Dance Revolution (DDR) video game study, in which an active video game was found to promote physical activity and decrease sedentary screen time [57], and from an indoor recess dance video study that showed dance videos might be an effective method for increasing school children’s PA during indoor recess as an alternative to sedentary activities [58]. However, children in both the aforementioned studies were slightly older than those in our study.

In the current study, we found only weak relationships between the mothers’ and children’s objectively measured SB and PA. This result is partly supported by Jago et al. (2010) who reported that there is a relationship between the objectively measured sedentary time of parents and their 10–11-year-old daughters, but not sons [59]. There was no relationship between parents’ and children’s time engaged in PA [59]. Contrary to our results, Abbott et al. (2016) found that both mothers’ and fathers’ PA were associated with the PA among 3–5-year-old girls, but not boys [26]. However, the children of that study were younger than those in the present study. Furthermore, Abbott et al.’s study was cross-sectional and only the children’s PA was assessed via accelerometers while the parents’ PA was self-reported. Cantell et al. (2012) reported a non-significant correlation between the children’s and mothers’ objectively measured MVPA [60], which is line with our study, even if children were on average younger than in our study. They also found that the best predictors of higher MVPA on weekdays were the child being older and the mothers’ greater time spent in organized PA, while during weekends the father’s role was more important than the mother’s [60].

In the present study, mothers (regardless of the group) reported that they had an average screen time of slightly more than two hours per day, and the children had an average screen time of around one and half hours per day. A greater proportion of mothers in the intervention group exceeded the two-hour screen time recommendations at the end of the intervention. Given that the intervention was screen-based, it is possible that this had an effect on the mothers’ screen time. However, mothers reported on average less exercise and a shorter duration of exercise time at the end of the study. The children’s results are in line with the Finland’s latest Report Card (2016), which reported that during weekdays 97% of children under school age (7 years in Finland) use media devices for a maximum of two hours per day; the corresponding figure for the weekends is 83% [41]. We did not find changes in the children’s self-reported screen time in the intervention group, although we had a screen-based intervention. In self-reported screen time, we found a moderate correlation between the mothers and children, and the correlation seemed to be strongest between the control group boys and mothers. Jago et al. (2014) reported a strong relationship between the parent’s and child’s screen-viewing, and they showed that the patterns were different for weekdays and the weekend [61].

In the supplementary analysis, we found that when the mother’s BMI was higher, she had more sedentary time. However, when there was a one-unit increase in BMI, the difference in SB was only a few minutes when expressed in minutes per day. In addition, whether the mother worked outside the home or not had an effect on SS, with the standing time being around 18 min/d longer among working mothers. A clinical or health-related mean of the difference needs further study. Children who stayed at home instead of attending daycare or preschool had on average more sedentary time and less physical activity. Although the earlier studies have shown that the activities in daycare are sedentary in nature [39], this study showed that activities might be even more sedentary at home. In the current study, we suggest that higher PA outside of home may result from having more friends to play with, or walking from home to daycare. It has been evaluated that 70% of comprehensive school students in Finland actively commute to school [41], but more information is needed about active transportation to daycare and preschool among younger children. Thus, these results are a guide to target physical activity interventions not only at daycare and preschool but at home and transportation, too.

The results and small changes over time in the current study might be related to the attractiveness of the video in relation to the age and gender of children. The study was planned primarily for slightly younger children, about 5 years of age, and in the present study’s children were on average 6.5 years old. We also think, based on the open questionnaire comments, that the younger children of the participating families liked the video more than the child who was involved to the study. However, the mothers’ and children’s comments and opinions of the movement-to-music video need to be analyzed in more detail before further conclusions can be drawn. In addition, it is possible that the duration and intensity of exercise within the movement-to-music video program was not vigorous enough to indicate changes in PA.

The lack of statistically significant results might also be related to maintaining motivation over the eight-week period. The intervention group was asked to perform the same three video sessions repeatedly without any variety. Partly because of the reason mentioned earlier (child’s age, the lack of variety), eight weeks might be too long for the use of a video without any interactive function or other motivating action. A review by Biddish and Irwin (2010) reported that active video games could promote light-to-moderate PA among children and young people [62]. Most of the reviewed studies were, however, conducted among children/young people only, without the parents’ active role in these studies, and most of these children were older than the children in our study. Further, in our study some of those mother-child pairs who reported using the video during week 8 reported that not only the mother-child pair but also the whole family performed the exercises. The way in which some families motivate themselves over eight weeks is in line with Paez et al. (2009), who found that parental and peer participation may play a role in children’s initial and sustained participation in PA studies [63]. In addition, based on the mothers’ reports, over time some children discovered their own movements and figures to music instead of the instructed ones included in our study.

### Strengths and limitations of the study

To our knowledge, this is the first study examining objectively measured SB and PA using a movement-to-music video program in the home environment at the same time among mothers and their children. The major strength of the study is the RCT design and the use of valid [45] and feasible [22] tri-axial accelerometer for measurements. Lying and sitting could be reliably separated from standing [46], which produced more detailed knowledge, not only for PA but specifically, for SB. However, we should also discuss the nature of hip-worn accelerometers, which are likely to underestimate shaking movements and jiggling of the hands and/or legs during the movement-to-music video program. Nevertheless, the accelerometer is able to detect overall PA and SB [44], which was the main focus of the study. The mean wearing times per day (13.3 h/d among children and 14.6 h/d among mothers) were similar to those reported in the other studies [31,50], and the participants had on average 6.0–6.7 valid days per week, which increases the reliability of the measured SB and PA. Finally, more than 80% of the included (n = 203) mother-child pairs participated until the end of the study, which is a high participation rate.

The primary weakness of our study is that our video was aimed at younger children than those involved. The above-mentioned difficulties in maintaining motivation over eight weeks because of the lack of variety possibly undermined the effect of the movement-to-music video. Since we know how important a role motivation plays for less SB and more PA, it would be interesting to study the effect of the current video among younger children, or to study the effect of various videos or some other kind music activities with the same age group. In earlier studies, music-related interventions have been shown to be effective in both adults [8,18] and children [19,21,22].

In our study, mothers were asked to report their own and their child’s exercise adherence in diaries during weeks 1, 2, and 8. They were also asked to evaluate on average how many times per week and for how long they had exercised during the whole intervention period. However, the second weakness of our study is that the difference in exercise adherence reported in the eight week diaries and questionnaire was essential. An additional limitation could be few diaries which were completed showing mothers and children had exercised using the video during week eight. In order to ensure compliance, we should have had the mothers keep diaries during the whole intervention period.

The third weakness is related to the cohort. The mothers who participated in the NELLI 7-year follow-up study might be more active and more aware of healthier lifestyles than the average women. This may have biased the results and reduced their generalizability, and may further partly explain the small changes over time within and between groups.

## Conclusion

The movement-to-music video program did not change objectively measured SB or PA. However, mothers and children seem to be more sedentary at home than at work and preschool or daycare, and therefore, interventions to decrease SB and increase PA should be targeted especially at the home environment. In addition, for those mothers and young children who have difficulties in exercising outside the home, the movement-to-music video program might represent a way to be physically active. With regard to children’s and parent’s SB and PA, more high-quality randomized controlled trials are needed to examine the effect of music-based exercise programs. With the limitations mentioned above, our study was pioneering for this purpose.

## Supporting information

S1 Table Change within and between groups of mothers in sedentary behavior and physical activity over time as a proportion of measurement time (estimates, 95% confidence intervals and *p*-value). Including mothers (n = 130) who had acceptable accelerometer use for all three weeks.
(PDF)

S2 Table Change within and between groups of children in sedentary behavior and physical activity over time as a proportion of measurement time (estimates, 95% confidence intervals and *p*-value). Including children (n = 111) who had acceptable accelerometer use for all three weeks.
(PDF)

S3 Table Change within and between groups of mothers in sedentary behavior and physical activity over time as a proportion of measurement time (estimates, 95% confidence intervals, and *p*-values). Including those mothers who used the movement-to-music video program (based on diaries, n = 9) at week 8 and those who belonged to the control group (n = 96).
(PDF)

S4 Table Change within and between groups of children in sedentary behavior and physical activity over time as a proportion of measurement time (estimates, 95% confidence intervals, and *p*-values). Including those children who used the movement-to-music video program (based on diaries, n = 10) at week 8 and those who belonged to the control group (n = 91).
(PDF)

S1 Text CONSORT 2010 checklist. (PDF)

S2 Text Research proposal for Ethics Committee of the human sciences. (PDF)

## Abbreviations

BMI: body mass index
CI: confidence interval
LME: linear mixed effects model
LPA: light physical activity
MET: metabolic equivalent
MVPA: moderate-to-vigorous physical activity
PA: physical activity
RCT: randomized controlled trial
SB: sedentary behavior
SD: standard deviation
SS: standing still
VAS: visual analog scale

## References

[1] de Rezende LF, Rodrigues Lopes M, Rey-Lopez JP, Matsudo VK, Luiz Odo C. Sedentary behavior and health outcomes: an overview of systematic reviews. PLoS One 2014 8 21;9(8):e105620 doi: 10.1371/journal.pone.0105620 25144686 10.1371/journal.pone.0105620 PMC4140795

[2] LeBlanc AG, Spence JC, Carson V, Connor Gorber S, Dillman C, Janssen I, et al Systematic review of sedentary behaviour and health indicators in the early years (aged 0–4 years). Appl Physiol Nutr Metab 2012 8;37(4):753–772. 22765839 10.1139/h2012-063

[3] Tremblay MS, LeBlanc AG, Kho ME, Saunders TJ, Larouche R, Colley RC, et al Systematic review of sedentary behaviour and health indicators in school-aged children and youth. Int J Behav Nutr Phys Act 2011 9 21;8:98-5868-8-98. 10.1186/1479-5868-8-98 PMC3186735 21936895

[4] Janssen I, Leblanc AG. Systematic review of the health benefits of physical activity and fitness in school-aged children and youth. Int J Behav Nutr Phys Act 2010 5 11;7:40-5868-7-40. 10.1186/1479-5868-7-40 PMC2885312 20459784

[5] Physical Activity Guidelines Advisory Committee. Physical activity guidelines advisory committee report, 2008 Washington, DC: U.S. Department of Health and Human Services Retrieved from http://www.health.gov/paguidelines/. 2008.

[6] Chtourou H, Jarraya M, Aloui A, Hammouda O, Souissi N. The effects of music during warm-up on anaerobic performances of young sprinters. Science & Sports 2012 12;27(6):e85–e88.

[7] Savitha D, Mallikarjuna RN, Rao C. Effect of different musical tempo on post-exercise recovery in young adults. Indian J Physiol Pharmacol 2010 Jan-Mar;54(1):32–36. 21046917

[8] Karageorghis CI. Applying music in exercise and sport Champaign, IL. London, UK: Human Kinetics; 2016.

[9] Biagini MS, Brown LE, Coburn JW, Judelson DA, Statler TA, Bottaro M, et al Effects of Self-Selected Music on Strength, explosiveness, and mood. Journal of Strength & Conditioning Research (Lippincott Williams & Wilkins) 2012 07;26(7):1934–1938. 10.1519/JSC.0b013e318237e7b3 22033366

[10] Barwood MJ, Weston NJV, Thelwell R, Page J. A motivational music and video intervention improves high-intensity exercise performance. Journal of Sports Science and Medicine 2009;8(3):435–442. 24150008 PMC3763290

[11] Bood RJ, Nijssen M, van der Kamp J, Roerdink M. The Power of Auditory-Motor Synchronization in Sports: Enhancing Running Performance by Coupling Cadence with the Right Beats. Plos One 2013 8 7;8(8):e70758 doi: 10.1371/journal.pone.0070758 23951000 10.1371/journal.pone.0070758 PMC3737354

[12] Atkinson G, Wilson D, Eubank M. Effects of music on work-rate distribution during a cycling time trial. Int J Sports Med 2004 11;25(8):611–615. doi: 10.1055/s-2004-815715 15532005 10.1055/s-2004-815715

[13] Bacon CJ, Myers TR, Karageorghis CI. Effect of music-movement synchrony on exercise oxygen consumption. J Sports Med Phys Fitness 2012 8;52(4):359–365. 22828457

[14] Zaletel P, Gabrilo G, Peric M. The training effects of dance aerobics: A review with an emphasis on the perspectives of investigations. Coll Antropol 2013 5;37 Suppl 2:125–130. 23914499

[15] Karageorghis CI, Priest DL, Williams LS, Hirani RM, Lannon KM, Bates BJ. Ergogenic and psychological effects of synchronous music during circuit-type exercise. Psychol Sport Exerc 2010 11 2010;11(6):551–559.

[16] Karageorghis CI, Priest DL. Music in the exercise domain: a review and synthesis (Part I). Int Rev Sport Exerc Psychol 2012 3;5(1):44–66. doi: 10.1080/1750984X.2011.631026 22577472 10.1080/1750984X.2011.631026 PMC3339578

[17] Karageorghis CI, Priest DL. Music in the exercise domain: a review and synthesis (Part II). Int Rev Sport Exerc Psychol 2012 3;5(1):67–84. doi: 10.1080/1750984X.2011.631027 22577473 10.1080/1750984X.2011.631027 PMC3339577

[18] Clark IN, Baker FA, Taylor NF. The modulating effects of music listening on health- related exercise and physical activity in adults: a systematic review and narrative synthesis. Nordic Journal of Music Therapy 2016 1 2;25(1):76–104.

[19] Dieringer ST, Porretta D, Gumm E. Using Music Therapy Principles to Enhance Physical Activity Participation In Children and Adolescents With Disabilities. Palaestra 2013 09;27(3):42–46.

[20] Denac O. A Case Study of Preschool Children's Musical Interests at Home and at School. Early Childhood Education Journal 2008 4 2008;35(5):439–444.

[21] Ward DS, Vaughn A, McWilliams C, Hales D. Interventions for increasing physical activity at child care. Med Sci Sports Exerc 2010 3;42(3):526–534. doi: 10.1249/MSS.0b013e3181cea406 20068495 10.1249/MSS.0b013e3181cea406

[22] Tuominen PP, Husu P, Raitanen J, Luoto RM. Differences in sedentary time and physical activity among mothers and children using a movement-to-music video program in the home environment: a pilot study. Springerplus 2016 1 28;5:93-016-1701-z. eCollection 2016. 10.1186/s40064-016-1701-z PMC4729752 26848433

[23] Jago R, Sebire SJ, Edwards MJ, Thompson JL. Parental TV viewing, parental self-efficacy, media equipment and TV viewing among preschool children. Eur J Pediatr 2013;172(11):1543–1545. doi: 10.1007/s00431-013-2077-5 23812514 10.1007/s00431-013-2077-5

[24] O'Dwyer MV, Fairclough SJ, Knowles Z, Stratton G. Effect of a family focused active play intervention on sedentary time and physical activity in preschool children. Int J Behav Nutr Phys Act 2012 10 1;9:117-5868-9-117. 10.1186/1479-5868-9-117 PMC3495835 23025568

[25] Xu H, Wen LM, Rissel C. Associations of parental influences with physical activity and screen time among young children: a systematic review. J Obes 2015;2015:546925 doi: 10.1155/2015/546925 25874123 10.1155/2015/546925 PMC4383435

[26] Abbott G, Hnatiuk J, Timperio A, Salmon J, Best K, Hesketh KD. Cross-sectional and Longitudinal Associations Between Parents' and Preschoolers' Physical Activity and Television Viewing: The HAPPY Study. J Phys Act Health 2016 3;13(3):269–274. doi: 10.1123/jpah.2015-0136 26181513 10.1123/jpah.2015-0136

[27] McMurray RG, Berry DC, Schwartz TA, Hall EG, Neal MN, Li S, et al Relationships of physical activity and sedentary time in obese parent-child dyads: a cross-sectional study. BMC Public Health 2016 2 6;16:124-016-2795-5. 10.1186/s12889-016-2795-5 PMC4744403 26851940

[28] Rebold MJ, Lepp A, Kobak MS, McDaniel J, Barkley JE. The Effect of Parental Involvement on Children's Physical Activity. J Pediatr 2016 3;170:206–210. doi: 10.1016/j.jpeds.2015.11.072 26725460 10.1016/j.jpeds.2015.11.072

[29] Dumuid D, Olds TS, Lewis LK, Maher C. Does home equipment contribute to socioeconomic gradients in Australian children’s physical activity, sedentary time and screen time? BMC Public Health 2016;16(1):736. 27496020 10.1186/s12889-016-3419-9 PMC4975892

[30] Spittaels H, Van Cauwenberghe E, Verbestel V, De Meester F, Van Dyck D, Verloigne M, et al Objectively measured sedentary time and physical activity time across the lifespan: a cross-sectional study in four age groups. Int J Behav Nutr Phys Act 2012 12 18;9:149-5868-9-149. 10.1186/1479-5868-9-149 PMC3542099 23249449

[31] Husu P, Suni J, Vaha-Ypya H, Sievanen H, Tokola K, Valkeinen H, et al Objectively measured sedentary behavior and physical activity in a sample of Finnish adults: a cross-sectional study. BMC Public Health 2016 9 1;16:920-016-3591-y. 10.1186/s12889-016-3591-y PMC5009485 27586887

[32] Recommendations for physical activity in early childhood 2016. Joy, play and doing together Ministry of Education and Culture 2016;21:Finland, 2016.

[33] Tremblay MS, Leblanc AG, Carson V, Choquette L, Connor Gorber S, Dillman C, et al Canadian Physical Activity Guidelines for the Early Years (aged 0–4 years). Appl Physiol Nutr Metab 2012 4;37(2):345–369. doi: 10.1139/h2012-018 22448608 10.1139/h2012-018

[34] Australian Government, Department of Health. Make your move—sit less, be active for life! Australia's physical activity and sedentary behavior guidelines for 5–12 years. Commonwealth of Australia 2014.

[35] Australian Government, Department of Health and Ageing. Move and play every day: National physical activity recommendations for children 0–5 years Commonwealth of Australia: Department of Health and Ageing 2010.

[36] Department of Health, Physical Activity, Health Improvement and Protection. Start active, stay active: A report on physical activity for health from the four home countries' Chief Medical Officers. United Kingdom 2011.

[37] Hnatiuk JA, Salmon J, Hinkley T, Okely AD, Trost S. A Review of Preschool Children’s Physical Activity and Sedentary Time Using Objective Measures. Am J Prev Med 2014 10;47(4):487–497. doi: 10.1016/j.amepre.2014.05.042 25084681 10.1016/j.amepre.2014.05.042

[38] Tammelin TH, Aira A, Hakamaki M, Husu P, Kallio J, Kokko S, et al Results From Finland's 2016 Report Card on Physical Activity for Children and Youth. J Phys Act Health 2016 11;13(11 Suppl 2):S157–S164. 27848744 10.1123/jpah.2016-0297

[39] Soini A, Willberg J, Gubbels J, Mehtälä A, Kettunen T, Poskiparta M. Directly observed physical activity among 3-year-olds in Finnish childcare. IJEC 2014;46:253–269.

[40] Anne Soini. Always on the move? Measured physical activity of 3-year-old preschool childrenUniversity of Jyväskylä; 2015.

[41] Finland's Report Card 2016 on Physical Activity for Children and Youth. LIKES Research reports on physical activity and health 320. Jyväskylä: LIKES Research Centre for Physical Activity and Health 2016.

[42] Schulz KF, Altman DG, Moher D, CONSORT Group. CONSORT 2010 Statement: updated guidelines for reporting parallel group randomised trials. BMC Med 2010 3 24;8:18-7015-8-18. 10.1186/1741-7015-8-18 PMC2860339 20334633

[43] Tuominen PP, Husu P, Raitanen J, Luoto RM. Rationale and methods for a randomized controlled trial of a movement-to-music video program for decreasing sedentary time among mother-child pairs. BMC Public Health 2015 10 5;15:1016-015-2347-4. 10.1186/s12889-015-2347-4 PMC4595194 26438056

[44] Vaha-Ypya H, Vasankari T, Husu P, Suni J, Sievanen H. A universal, accurate intensity-based classification of different physical activities using raw data of accelerometer. Clin Physiol Funct Imaging 2015 1;35(1):64–70. doi: 10.1111/cpf.12127 24393233 10.1111/cpf.12127

[45] Vaha-Ypya H, Vasankari T, Husu P, Manttari A, Vuorimaa T, Suni J, et al Validation of Cut-Points for Evaluating the Intensity of Physical Activity with Accelerometry-Based Mean Amplitude Deviation (MAD). PLoS One 2015 8 20;10(8):e0134813 doi: 10.1371/journal.pone.0134813 26292225 10.1371/journal.pone.0134813 PMC4546343

[46] Sievänen H, Vähä-Ypyä H, Husu P, Suni J, Vasankari T. A universal method for accurate classification of physical activity and sedentary behavior with tri-axial accelerometry. Med Sci Sports Exerc 2014;46:S438.

[47] Sedentary Behavior Research Network. Standardized use of the terms "sedentary" and "sedentary behaviors". Appl Physiol Nutr Metab 2012;37:540–542. doi: 10.1139/h2012-024 22540258 10.1139/h2012-024

[48] Tremblay MS, Colley RC, Saunders RP, Healy GN, Owen N. Psysiological and health implications of a sedentary lifestyle. Appl Physiol Nutr Metab 2010;35(2):725–740. 21164543 10.1139/H10-079

[49] Aittasalo M, Vaha-Ypya H, Vasankari T, Husu P, Jussila AM, Sievanen H. Mean amplitude deviation calculated from raw acceleration data: a novel method for classifying the intensity of adolescents' physical activity irrespective of accelerometer brand. BMC Sports Sci Med Rehabil 2015 8 7;7:18-015-0010-0. eCollection 2015. 10.1186/s13102-015-0010-0 PMC4527117 26251724

[50] Husu P, Vaha-Ypya H, Vasankari T. Objectively measured sedentary behavior and physical activity of Finnish 7- to 14-year-old children- associations with perceived health status: a cross-sectional study. BMC Public Health 2016 4 16;16:338-016-3006-0. 10.1186/s12889-016-3006-0 PMC4833900 27083559

[51] Koskinen S, Lundqvist A, Ristiluoma N. Terveys, toimintakyky ja hyvinvointi Suomessa 2011. 2012;2012_068.

[52] Husu P, Paronen O, Suni J, Vasankari T. Suomalaisten fyysinen aktiivisuus ja kunto 2010. Terveyttä edistävän liikunnan nykytila ja muutokset. [Physical activity and fitness of Finns in 2010: The current status of, and changes in, health enhancing physical activity], with English summary. 2011;15/2011.

[53] Benjamins M, Hummer R, Eberstein I, Nam C. Self-reported health and adult mortality risk: An analysis of cause-specific mortality. Soc Sci Med (1982) 2004;59(6):1297–1306. 10.1016/j.socscimed.2003.01.001 15210100

[54] Mäki P, Laatikainen T, Koponen P, Hakulinen-Viitanen T. The development of health monitoring among children and the young, LATE project. 2008;B28.

[55] Kincaid C. Guidelines for selecting the covariance structure in mixed model analysis. In Proceedings of the Thirteenth Annual SAS Users Group International Conference April 2005;No. 198–30.

[56] Moher D, Hopewell S, Schulz KF, Montori V, Gøtzsche PC, Devereaux PJ, et al CONSORT 2010 Explanation and Elaboration: updated guidelines for reporting parallel group randomised trials. J Clin Epidemiol 2010 8;63(8):e1–e37. doi: 10.1016/j.jclinepi.2010.03.004 20346624 10.1016/j.jclinepi.2010.03.004

[57] Maloney AE, Carter Bethea T, Kelsey KS, Marks JT, Paez S, Rosenberg AM, et al A Pilot of a Video Game (DDR) to Promote Physical Activity and Decrease Sedentary Screen Time. Obesity 2008 9 2008;16(9):2074–80. doi: 10.1038/oby.2008.295 19186332 10.1038/oby.2008.295

[58] Erwin H, Koufoudakis R, Beighle A. Children's Physical Activity Levels During Indoor Recess Dance Videos. J Sch Health 2013 05;83(5):322–327 6p. doi: 10.1111/josh.12034 23516999 10.1111/josh.12034

[59] Jago R, Fox KR, Page AS, Brockman R, Thompson JL. Parent and child physical activity and sedentary time: do active parents foster active children? BMC Public Health 2010 4 15;10:194-2458-10-194. 10.1186/1471-2458-10-194 PMC2868817 20398306

[60] Cantell M, Crawford SG, Dewey D. Daily physical activity in young children and their parents: A descriptive study. Paediatr Child Health 2012 3;17(3):e20–4. 23450045 10.1093/pch/17.3.e20 PMC3287098

[61] Jago R, Thompson JL, Sebire SJ, Wood L, Pool L, Zahra J, et al Cross-sectional associations between the screen-time of parents and young children: differences by parent and child gender and day of the week. Int J Behav Nutr Phys Act 2014 4 23;11:54-5868-11-54. 10.1186/1479-5868-11-54 PMC4004449 24758143

[62] Biddiss E, Irwin J. Active video games to promote physical activity in children and youth: a systematic review. Arch Pediatr Adolesc Med 2010 7;164(7):664–672. doi: 10.1001/archpediatrics.2010.104 20603468 10.1001/archpediatrics.2010.104

[63] Paez S, Maloney A, Kelsey K, Wiesen C, Rosenberg A. Parental and environmental factors associated with physical activity among children participating in an active video game. Pediatr Phys Ther 2009 Fall;21(3):245–253. 19680066 10.1097/PEP.0b013e3181b13a82

