# Supplementary material for: The effect of a movement-to-music video program on the objectively measured sedentary time and physical activity of preschool-aged children and their mothers: A randomized controlled trial
Source: PLoS One. 2017 Aug 31;12(8):e0183317. doi: 10.1371/journal.pone.0183317 (PMC5578653; doi:10.1371/journal.pone.0183317)
Supplement: S1 Table — Including mothers (n = 130) who had acceptable accelerometer use for all three weeks. (PDF) [file pone.0183317.s001.pdf]

S1 Table. Change within and between groups of mothers in sedentary behavior and physical activity over time as a proportion of measurement time (estimates, 95% confidence intervals and p-value).

Including mothers (n=130) who had acceptable accelerometer use in all three weeks.

	Unadjusted		Adjusted*	
MOTHERS	estimate (95% CI)	*p*-value	estimate (95% CI)	*p*-value
Sedentary behavior**				
difference at baseline (ref = control)	1.63 (-1.21 to 4.47)	0.258	1.95 (-0.88 to 4.78)	0.175
change in time, control	0.008 (-0.017 to 0.033)	0.521	0.015 (-0.012 to 0.042)	0.267
change in time, intervention	-0.009 (-0.035 to 0.017)	0.488	-0.009 (-0.036 to 0.018)	0.514
intervention effect (ref = control)	-0.017 (-0.054 to 0.019)	0.346	-0.024 (-0.062 to 0.014)	0.214
Standing still**				
difference at baseline (ref = control)	-0.65 (-2.257 to 0.962)	0.427	-0.71 (-2.352 to 0.941)	0.398
change in time, control	-0.007 (-0.023 to 0.010)	0.435	-0.010 (-0.028 to 0.008)	0.275
change in time, intervention	0.011 (-0.007 to 0.028)	0.235	0.010 (-0.008 to 0.029)	0.272
intervention effect (ref = control)	0.017 (-0.007 to 0.041)	0.163	0.020 (-0.006 to 0.046)	0.122
Light physical activity**				
difference at baseline (ref = control)	-0.287 (-1.690 to 1.115)	0.686	-0.605 (-2.096 to 0.887)	0.424
change in time, control	-0.004 (-0.016 to 0.008)	0.530	-0.006 (-0.019 to 0.007)	0.366
change in time, intervention	-0.001 (-0.014 to 0.011)	0.815	-0.002 (-0.015 to 0.012)	0.813
intervention effect (ref = control)	0.002 (-0.015 to 0.019)	0.792	0.004 (-0.014 to 0.023)	0.641
Moderate-to-vigorous physical activity**				
difference at baseline (ref = control)	-0.693 (-1.667 to 0.281)	0.162	-0.635 (-1.639 to 0.370)	0.213
change in time, control	0.003 (-0.005 to 0.012)	0.447	0.002 (-0.008 to 0.012)	0.712
change in time, intervention	0.001 (-0.008 to 0.010)	0.881	0.0003 (-0.010 to 0.010)	0.956
intervention effect (ref = control)	-0.003 (-0.015 to 0.010)	0.677	-0.002 (-0.016 to 0.013)	0.826

* Adjusted for mother's BMI, number of children, work, disorders or symptoms, and perceived health

** Proportion of measurement time
