# Supplementary material for: The effect of a movement-to-music video program on the objectively measured sedentary time and physical activity of preschool-aged children and their mothers: A randomized controlled trial
Source: PLoS One. 2017 Aug 31;12(8):e0183317. doi: 10.1371/journal.pone.0183317 (PMC5578653; doi:10.1371/journal.pone.0183317)
Supplement: S2 Table — Including children (n = 111) who had acceptable accelerometer use for all three weeks. (PDF) [file pone.0183317.s002.pdf]

S2 Table. Change within and between groups of children in sedentary behavior and physical activity over time as a proportion of measurement time (estimates, 95% confidence intervals and p-value).

Including children (n=111) who had acceptable accelerometer use in all three weeks.

| | Unadjusted | | Adjusted* | |
|---|---|---|---|---|
| CHILDREN | estimate (95% CI) | *p*-value | estimate (95% CI) | *p*-value |
| Sedentary behavior** | | | | |
| difference at baseline (ref = control) | -0.191 (-2.612 to 2.230) | 0.876 | -0.099 (-2.513 to 2.315) | 0.935 |
| change in time, control | 0.013 (-0.013 to 0.040) | 0.329 | 0.016 (-0.011 to 0.043) | 0.251 |
| change in time, intervention | 0.016 (-0.013 to 0.044) | 0.283 | 0.015 (-0.013 to 0.043) | 0.280 |
| intervention effect (ref = control) | 0.002 (-0.037 to 0.041) | 0.902 | -0.0005 (-0.040 to 0.039) | 0.980 |
| Standing still** | | | | |
| difference at baseline (ref = control) | 0.108 (-0.723 to 0.940) | 0.797 | -0.103 (-0.951 to 0.745) | 0.810 |
| change in time, control | -0.001 (-0.011 to 0.010) | 0.907 | -0.003 (-0.013 to 0.008) | 0.606 |
| change in time, intervention | -0.005 (-0.016 to 0.006) | 0.400 | -0.004 (-0.015 to 0.006) | 0.424 |
| intervention effect (ref = control) | -0.004 (-0.019 to 0.011) | 0.591 | -0.002 (-0.017 to 0.013) | 0.828 |
| Light physical activity** | | | | |
| difference at baseline (ref = control) | -0.117 (-1.332 to 1.097) | 0.848 | -0.050 (-1.277 to 1.177) | 0.936 |
| change in time, control | -0.013 (-0.025 to -0.001) | **0.037** | -0.013 (-0.255 to -0.0002) | **0.046** |
| change in time, intervention | -0.014 (-0.027 to -0.001) | **0.041** | -0.014 (-0.027 to -0.001) | **0.038** |
| intervention effect (ref = control) | -0.001 (-0.019 to 0.017) | 0.934 | -0.001 (-0.019 to 0.017) | 0.916 |
| Moderate-to-vigorous physical activity** | | | | |
| difference at baseline (ref = control) | 0.197 (-1.225 to 1.620) | 0.784 | 0.247 (-1.156 to 1.650) | 0.728 |
| change in time, control | -0.001 (-0.016 to 0.015) | 0.948 | -0.001 (-0.017 to 0.015) | 0.887 |
| change in time, intervention | 0.002 (-0.014 to 0.019) | 0.784 | 0.002 (-0.014 to 0.019) | 0.765 |
| intervention effect (ref = control) | 0.003 (-0.020 to 0.025) | 0.807 | 0.004 (-0.019 to 0.026) | 0.754 |

* Adjusted for child's BMI, daycare or preschool, and number of siblings

** Proportion of measurement time
