# Supplementary material for: The effect of a movement-to-music video program on the objectively measured sedentary time and physical activity of preschool-aged children and their mothers: A randomized controlled trial
Source: PLoS One. 2017 Aug 31;12(8):e0183317. doi: 10.1371/journal.pone.0183317 (PMC5578653; doi:10.1371/journal.pone.0183317)
Supplement: S3 Table — Including those mothers who used the movement-to-music video program (based on diaries, n = 9) at week 8 and those who belonged to the control group (n = 96). (PDF) [file pone.0183317.s003.pdf]

S3 Table. Change within and between groups of mothers in sedentary behavior and physical activity over time as a proportion of measurement time (estimates, 95% confidence intervals and p-value).

Including those mothers who used movement-to-music video program (based on diaries, n=9) during the last week or belonged to the control group (n=96).

	Unadjusted		Adjusted*	
MOTHERS	estimate (95% CI)	*p*-value	estimate (95% CI)	*p*-value
Sedentary behavior**				
difference at baseline (ref = control)	-0.39 (-6.400 to 5.617)	0.898	2.48 (-3.799 to 8.755)	0.434
change in time, control	0.008 (-0.015 to 0.033)	0.504	0.015 (-0.011 to 0.042)	0.245
change in time, intervention	-0.008 (-0.077 to 0.061)	0.824	-0.006 (-0.079 to 0.067)	0.865
intervention effect (ref = control)	-0.016 (-0.089 to 0.057)	0.666	-0.022 (-0.099 to 0.056)	0.579
Standing still**				
difference at baseline (ref = control)	1.28 (-2.092 to 4.647)	0.454	1.21 (-2.427 to 4.854)	0.509
change in time, control	-0.008 (-0.024 to 0.009)	0.348	-0.011 (-0.029 to 0.006)	0.199
change in time, intervention	0.010 (-0.037 to 0.057)	0.664	-0.001 (-0.050 to 0.048)	0.972
intervention effect (ref = control)	0.018 (-0.032 to 0.068)	0.472	0.011 (-0.042 to 0.063)	0.688
Light physical activity**				
difference at baseline (ref = control)	0.331 (-2.480 to 3.143)	0.816	-1.35 (-4.368 to 1.676)	0.378
change in time, control	-0.003 (-0.015 to 0.009)	0.600	-0.005 (-0.018 to 0.008)	0.474
change in time, intervention	-0.006 (-0.041 to 0.028)	0.723	-0.0002 (-0.037 to 0.037)	0.992
intervention effect (ref = control)	-0.003 (-0.039 to 0.034)	0.873	0.005 (-0.035 to 0.044)	0.817
Moderate-to-vigorous physical activity**				
difference at baseline (ref = control)	-1.24 (-3.669 to 1.198)	0.316	-2.35 (-4.937 to 0.242)	0.075
change in time, control	0.003 (-0.006 to 0.012)	0.524	0.001 (-0.009 to 0.011)	0.827
change in time, intervention	0.004 (-0.021 to 0.030)	0.739	0.008 (-0.021 to 0.036)	0.592
intervention effect (ref = control)	0.001 (-0.026 to 0.028)	0.919	0.007 (-0.024 to 0.037)	0.666

* Adjusted for mother's BMI, number of children, work, disorders or symptoms, and perceived health

** Proportion of measurement time
