# Supplementary material for: The effect of a movement-to-music video program on the objectively measured sedentary time and physical activity of preschool-aged children and their mothers: A randomized controlled trial
Source: PLoS One. 2017 Aug 31;12(8):e0183317. doi: 10.1371/journal.pone.0183317 (PMC5578653; doi:10.1371/journal.pone.0183317)
Supplement: S4 Table — Including those children who used the movement-to-music video program (based on diaries, n = 10) at week 8 and those who belonged to the control group (n = 91). (PDF) [file pone.0183317.s004.pdf]

S4 Table. Change within and between groups of children in sedentary behavior and physical activity over time as a proportion of measurement time (estimates, 95% confidence intervals and p-value).

Including those children who used movement-to-music video program (based on diaries, n=10) during the last week or belonged to the control group (n=91).

	Unadjusted		Adjusted*	
CHILDREN	estimate (95% CI)	p-value	estimate (95% CI)	p-value
Sedentary behavior**				
difference at baseline (ref = control)	-0.928 (-4.930 to 3.073)	0.646	-0.411 (-4.271 to 3.449)	0.833
change in time, control	0.019 (-0.009 to 0.047)	0.173	0.023 (-0.005 to 0.052)	0.107
change in time, intervention	-0.023 (-0.096 to 0.044)	0.463	-0.028 (-0.097 to 0.040)	0.408
intervention effect (ref = control)	-0.045 (-0.120 to 0.030)	0.236	-0.052 (-0126 to 0.022)	0.166
Standing still**				
difference at baseline (ref = control)	0.230 (-1.330 to 1.790)	0.771	-0.092 (-1.647 to 1.463)	0.906
change in time, control	-0.003 (-0.013 to 0.008)	0.593	-0.005 (-0.016 to 0.006)	0.373
change in time, intervention	0.015 (-0.011 to 0.041)	0.259	0.016 (-0.010 to 0.042)	0.232
intervention effect (ref = control)	0.018 (-0.010 to 0.046)	0.213	0.021 (-0.008 to 0.047)	0.149
Light physical activity**				
difference at baseline (ref = control)	1.050 (-0.971 to 3.071)	0.305	0.866 (-1.199 to 2.931)	0.407
change in time, control	-0.015 (-0.027 to -0.003)	**0.018**	-0.015 (.0.027 to -0.002)	**0.020**
change in time, intervention	-0.005 (-0.035 to 0.024)	0.717	-0.005 (-0.035 to 0.025)	0.156
intervention effect (ref = control)	0.009 (-0.023 to 0.041)	0.567	0.010 (-0.022 to 0.042)	0.541
Moderate-to-vigorous physical activity**				
difference at baseline (ref = control)	-0.345 (-2.817 to 2.127)	0.782	-0.359 (-2.731 to 2.013)	0.764
change in time, control	-0.002 (-0.018 to 0.014)	0.785	-0.004 (-0.020 to 0.013)	0.676
change in time, intervention	0.017 (-0.023 to 0.057)	0.408	0.018 (-0.022 to 0.058)	0.369
intervention effect (ref = control)	0.019 (-0.024 to 0.062)	0.385	0.022 (-0.022 to 0.065)	0.323

* Adjusted for child's BMI, daycare or preschool, and number of siblings

** Proportion of measurement time
