# Supplementary material for: The effect of a movement-to-music video program on the objectively measured sedentary time and physical activity of preschool-aged children and their mothers: A randomized controlled trial
Source: PLoS One. 2017 Aug 31;12(8):e0183317. doi: 10.1371/journal.pone.0183317 (PMC5578653; doi:10.1371/journal.pone.0183317)
Supplement: S2 Text — (PDF) [file pone.0183317.s006.pdf]

Moving Sound – Experimental study on music and sedentariness among mothers and children, RCT

(Liikuttava sävel - TUTKIMUS MUSIIKKILIIKUNTAVIDEON VAIKUTUKSISTA ÄITIEN JA LASTEN FYYSISEEN AKTIIVISUUTEEN, *RCT)*

1. Theoretical background and the benefits of research

Risk for many chronic diseases, such as diabetes, obesity, breast cancer and cardiovascular diseases might be thorough impacted by an unhealthy maternal metabolic state (Murphy et al. 2006, Hilakivi-Clarke et al. 2006). A high birth weight of newborn has a harmful effect to next generation furthering a vicious transgenerational cycle of obesity and cardio-metabolic disorders (Cnattingius et al. 2012, Figure 1.). In randomized controlled study NELLI (Lifestyle, counselling and exercise in maternity care -project) inserted lifestyle counselling produced positive changes both in diet and physical activity (Kinnunen et al. 2007, Aittasalo et al. 2008). For women who have a risk for gestational diabetes mellitus, lifestyle counselling is effective in decreasing birth weight of the newborn (Luoto et al. 2011). There were also differences in one-year follow-up study between offspring of the women who were in intervention group and those who had got standard maternity care. Data from that study shows that offspring of the mothers who had received intensive dietary and physical activity counselling were less obese than offspring in the control group (Mustila et al. 2012).

Figure 1. Vicious cycle of transgenerational obesity – NELLI main cohort and current study Moving Sound. Adopted from Luoto, research proposal of NELLI 5-years follow-up study.

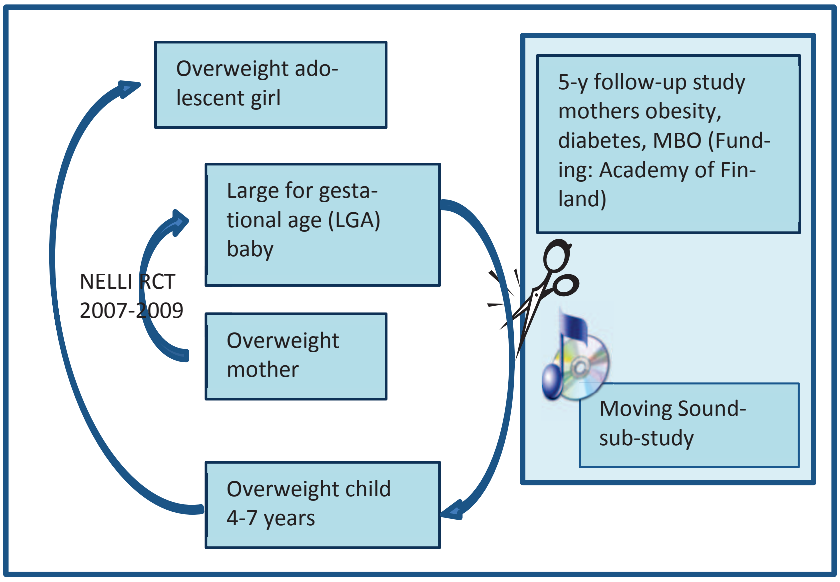

Transgenerational cycle of obesity and cardio-metabolic disorders should be restudied and prevented by decreasing sedentariness among both mother and offspring. There is a need to establish a two-part project, which is described in the Figure 1 above. A follow-up study for lifestyle intervention participants will aim to determine prevalence of diabetes, metabolic syndrome and obesity among mothers.

A sub-study entitled *Moving sound* will aim to cut the vicious cycle of transgenerational obesity and sedentariness by introducing motivating movement-to-music video programs for mother-child pairs. Participants to the Moving sound -study will be recruited from the cohort of the randomized controlled trial NELLI,

which was collected during the years 2007-2009. The protocol and methods of NELLI -study have been reported in detail previously (Luoto et al. 2010).

Music and exercise. Listening to music might have an ergogenic effects during aerobic exercise. The use of music decreases ratings of perceived exertion during exercise (Potteiger et al. 2000, Karageorghis et al. 2006, Yamasita et al. 2006) and it motivates to exercise at higher power (Atkinson et al. 2004). Music encourages participation in exercises and activities, and it helps in keeping the beat (Mathews et al. 2001, Murrock 2002, Hamburg & Clair 2003). Music may ease pain and help on adherence to a physical training program (Murrock 2002, Siedliecki & Good 2006). The use of music is able to reduce anxiety and stress, and it improves efficiency of relaxation techniques (Cevasco et al. 2005, Siedliecki & Good 2006). The conceptual framework for benefits of music is shown below in Figure 2.

Figure 2. Conceptual framework for benefits of music in sport and exercise contexts. Adapted from Terry & Karageorghis 2006 and Karageorhis & Priest 2012.

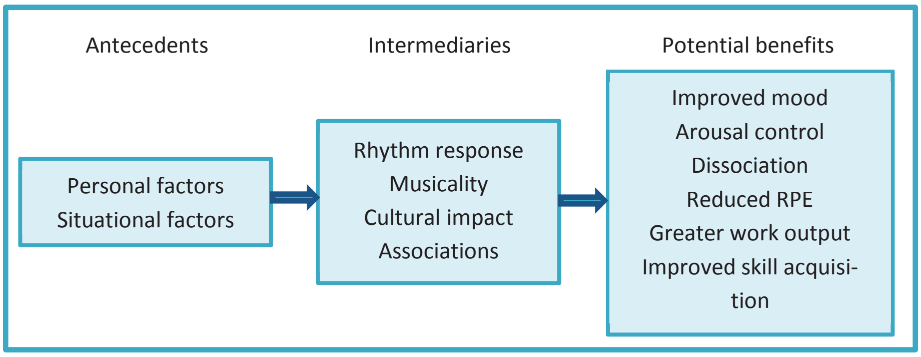

Musical elements (i.e. rhythm, tempo, beat, musical shape and form, melody, harmony, sound, dynamics, lyrics, and style) have an effect on an individual's impression of music (Karageorghis et al. 2006). Personal factors like gender, age, and engagement to exercise and situational factors like context of music and exercise appears to have an influence to a personal experiences (Karageorghis et al. 2012). It seems that the most important elements of the motivational qualities of music are fast tempo and rhythmic elements (Atkinson et al. 2004, Karageorghis et al. 2006). Not only tempo or rhythm but a musical ensemble (rhythmic and melodic elements, the listener's musical talent and extra-musical associations) motivates to exercise better than tempo and rhythm alone (Potteiger et al. 2000, Crust & Clough 2006, Karageorghis et al. 2006).

Listening to music might influence to an intensity of physical activity, it might have an effect on the rate of perceived exertion and general mood, and it might help to extend workouts (Karageorghis et al. 2012). In this way, music may improve exercise adherence by increasing motivation. In addition to the above described psychological benefits, also ergogenic i.e. work-enhancing effects such as increase of work capacity have been shown (Karageorghis 2008).

Overall, the benefits of music have been studied for therapeutic purposes and athlete's studies. There are no studies available using mother-child -pairs and/or motivational music in decreasing sedentariness. Only a few studies have examined in combination of the ergogenic effect of motivational music and video interventions. Such as music, video watching has a potential effect to shift attention from internal stimuli to external cues and motivational music with video enlarge the positive effects of audiovisual interventions (Barwood et al. 2009). Increasing physical activity and decreasing sedentary behavior it's possible to prevent and improve the following health problems caused by inactivity and achieve long term health benefits. From this perspective, the use of music and video together can give an added benefit.

Physical activity and sedentary behavior. Plenty of observational evidence shows that sedentary behavior is a distinct risk factor, independent of physical activity, for multiple health outcomes (Thorp et al. 2011, Lee et al. 2012) and mortality (Lee et al. 2012, Katzmarzyk et al. 2009, Patel et al. 2010). It may even be more strongly associated with cardio-metabolic risk factors than physical activity (Celis-Morales et al.

2012). In this study ‘sedentary behavior’ means sitting or reclining posture (very low energy expenditure) while waking hours and ‘inactivity’ means the lack of physical activity compared with physical activity guidelines (Sedentary Behavior Research Network 2012).

Self-reporting have been the most widely used methods to assess both sedentary behavior and physical activity. However they require participants to memorize and continuously record their activities. Additionally, validity and reliability of these self-reports is quite poor (van Poppel et al. 2010, Hagstromer et al. 2010, Clark et al. 2009). Moderate-to-vigorous physical activity is reported more reliably and validly than less intensive activities. The least reliably reported are low intensity activities, often performed in several short bouts within a day (Matthews et al. 2012).

During the last ten years technological development has produced new aspects for measurement of both physical activity and sedentariness. Tri-axial (3D) accelerometer, collecting information on raw mode instead of proprietary units, is becoming the method-of-choice in studies where accurate measurement is of primary importance (Freedson et al. 2012, Intille et al. 2012, Bassett et al. 2012).

2. Objectives and hypotheses

Quite many people use music during exercise for example while jogging or in the gym. Anyhow some mothers might have difficulties to go to move when they have small children and thereby importance of physical activity at home increases. In summary, there is a need to perform an experimental study which combines the understanding of the benefits of music, objective physical activity measurement in decreasing sedentariness and thereafter transgenerational obesity.

Main objective of the current study is to decrease sedentary behavior and increase physical activity among mothers and their offspring by using music-to-movement video program.

Primary outcomes of the study are objectively assessed sedentary behavior and physical activity of mothers and their children aged 4–7 years. Measurements will be done during the first, second and eighth weeks of the intervention. Sedentary behavior and physical activity time will be analyzed both in minutes and as a proportion of measurement time.

Secondary outcomes of the study are quantity and quality of self-reported screen time among mothers and children and motivational quality of music and music-to-movement video. Additional secondary outcomes are mother’s weight, workability, mental health and mood.

Specific aim is to study effectiveness of intervention with accelerometer use only or with a combination of accelerometer plus music-to-movement video program for mother-child pairs.

Specific hypotheses

I. Movement-to-music video program developed for mother-child pairs decrease sedentary time more than wearing accelerometer only. There are no changes in moderate-to-vigorous physical activity, although amount of light intensity physical activity increases.

II. Movement-to-music video motivates mother-child pairs increase physical activity and decrease sedentary behavior.

III. Mothers’ musicality motivates them exercise more with video program than mothers’ without musical background.

3. Research methods and materials

Accelerometer measures and pretest. Main aim of the project is to reduce sedentariness among women and their children. Accelerometer measures continuously tri-axial acceleration caused by any movement and permits accurate individual assessment of both physical activity and sedentary behavior. Data on physical activity and sedentary behavior will be collected with tri-axial accelerometer (Hookie AM 20, Traxmeet

Ltd, Espoo, Finland). The data will be collected in raw mode and analyzed as the mean signal amplitude deviation (MAD) of acceleration (Vähä-Ypyä et al. 2014), which permits accurate individual assessment of both physical activity and sedentariness. The algorithms used in the present study are based on pilot studies conducted at the UKK Institute.

Accelerometers are attached to a flexible belt and all women and their children will be instructed to wear the belt around their hip for 14 consecutive days (two weeks) during waking hours at the beginning of the study and 7 days (one week) at the last week of intervention time. The accelerometers should be removed before going to bed and during showers, bathing, swimming or other water activities.

Accelerometers were pretested with eleven 2–9 years old children. Children did different kind of free movements, play, and games in test track field during one hour. That was done to find out how scurry-like movements appear in accelerometers. Another test in running track tested different speeds from the slow walking to the maximum rate of running. In addition accelerometers piloted with ten 4–7 years children during one week. On the basis of these tests acceleration signal acted as expected.

***Movement-to-music DVD production and pretest**. .* There will be two separate movement-to-music video programs prepared by the Sibelius-Academy music education students in the specific course for children's music programs during spring 2014. Directors, Soili Perkiö and Eeva-Leena Pokela are children's music professionals. The student group is called Mutaveijarit (The Mud Mates). The Mud Mates "*live in puddles, ditches, ponds, creeks, and on the shores of oceans and lakes. They are hard to find, but by singing and playing, each of us, child and adult, can dive into their hilarious world. These are songs by the water—splashing, dipping, playing, and chattering.*"[1]

Video programs were produced for this study. Both videos last about 10 minutes including two songs and their movement preparation. There are three songs altogether, because the title song Mutaveijarit is in both tracks (Table 1.).

Table 1. Details of music used in movement-to-music DVD

Name of the song	Compose, lyrics and arrangement	Musical genre	Tempo (beats per minute)	Motivational quality video vs. music group (BMRI-2, max. 42 points)
**Video 1: Mutaveijarit ja karibialainen kala**				
Mutaveijarit	Eeva-Leena Pokela and Mutaveijarit	Rock'n'roll	94	34,6 vs. 33,7
Karibialainen kala	Aili Järvelä	Swing'n'Boogie	128	34,0 vs. 31,9
**Video 2: Kuraa ja mutaa**				
Kuravelli	Miia Reko and Mutaveijarit	Schottische	124	32,5 vs. 31,4
Mutaveijarit	Eeva-Leena Pokela and Mutaveijarit	Rock'n'roll	94	

To rate the motivational qualities of these three songs, a panel of 8 physiotherapists (all female) assessed each song by using Brunel Music Rating Inventory-2 (BMRI-2: Karageorghis, Priest et al. 2006; BMRI-3: Karageorghis 2008). Another reason for pretest was to find out the influence of visual stimuli on the responses to the music. BMRI-2 was translated in Finnish by investigators of this study.

Each song was rated by indicating one number between 1 (strongly disagree) and 7 (strongly agree) for six statements about how much the song would motivate a person during exercise. The possible range of scores is 6–42, wherein scores below 24 mean low/oudeterous, middle range 24–35 moderately and scores over 36 mean highly motivating ratings (Karageorghis 2008).

Video group (n=4, mean age 41 years, SD 16,2 years) was first watching DVD and assessed all three songs separately using BMRI-2. Then they listened only music (without illustration) and rated the motivational qualities of each song. Music group (n=4, mean age 42,8 years, SD 15,8 years) assessed first only the motivational qualities of the songs. After that music group was watching DVD and appraised the music and illus-

[1] Citation from event description http://www.musiikkitalo.fi/en/event/mutaveijarit-0

tration together. In addition both groups moved to DVD and rated the motivational qualities of music during movement.

The rhythm, style, melody, tempo and beat motivated movement more in video group than in music group. Music group assessed sound motivated them more than sound did in video group. During movement rhythm, style, and beat motivated video group more while melody, tempo, and sound motivated music group. As expected in both groups music and picture together motivated movement more than music alone.

Data collection. Objective measurements of sedentary behavior and physical activity of the mothers and their children will be collected at first, second and eighth weeks of the intervention by using accelerometer. Body weight of the mothers will be measured at baseline. Information on current physical activity, screen time, diet, and self-reported weight, as well as information on quality of life will be collected at the baseline by using same questionnaires as in the NELLI 5-years follow-up study (based on one-year follow-up questionnaires of the NELLI cohort). At 2 and 8 weeks after baseline information on physical activity, screen time, self-reported weight and motivation to exercise by using movement-to-music video program will be collected by questionnaires (Table 2). Information of the children's physical activity and screen time will be collected at baseline, 2nd and 8th weeks after baseline by questionnaires.

Table 2. Moving sound data collection and measurements at baseline, 2 weeks and 8 weeks after baseline.

Data collection	Baseline	2 weeks	8 weeks
Accelerometer measures	X	X	X
Physical activity questionnaire	X	X	X
Sedentary/screen time questionnaire	X	X	X
Weight	X	X	X
Motivation to exercise with video program		X	X
Mother's musical background (intervention group)		X	

Participants of the Moving Sound -study will be recruited from the cohort of the original NELLI-RTC. Mother and child -pairs (n=837) will be invited to NELLI 5-years follow-up study and to Moving Sound -study from 14 municipalities of Pirkanmaa area. We expect to get involved 30–40 % of the original cohort, which will be 251–335 mother and child -pairs, altogether. Possible reasons for drop out at this stage will be that a person can't be invited, because it's not possible to get contact for her, a person has moved away from the area or a person doesn't want to take part to study. Estimation for ***sample size and randomization of the study*** are shown in Figure 3.

Figure 3. Estimation for sample size and randomization of the study.

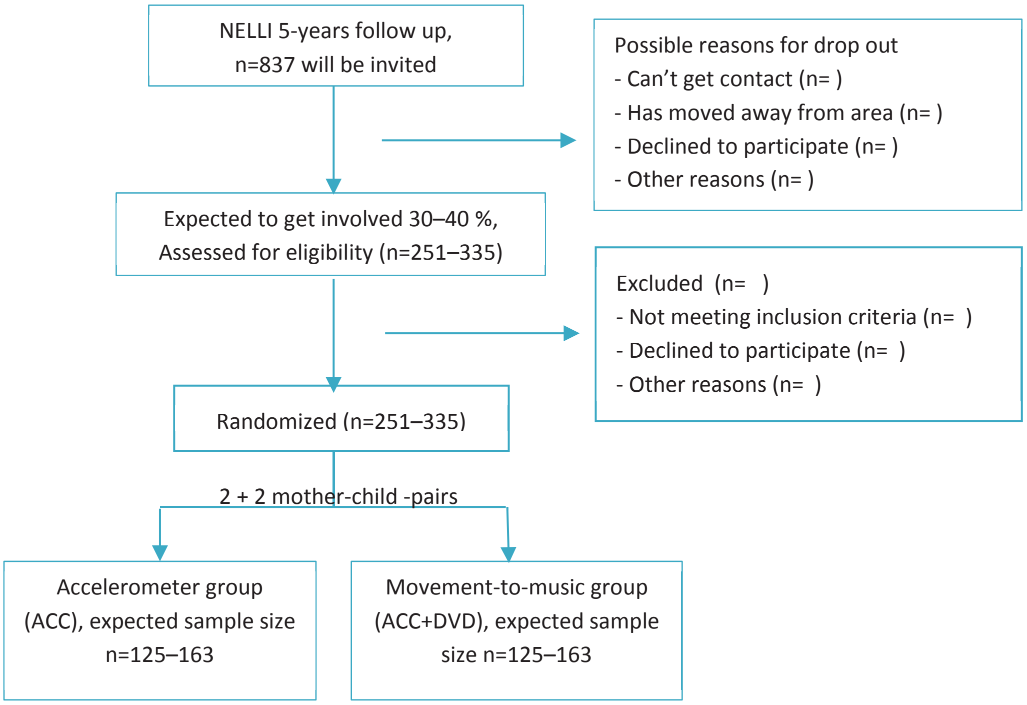

Mothers and children will be eligible for inclusion if they meet the following criteria: child age between 4 and 7 years, have a possibility to use DVD player or YouTube-link, and both of them will be able to perform physical activity and use accelerometer as instructed. Mothers and children who are unable to perform physical activity for medical reasons will be excluded.

Randomization will be done for 2:2 ratios, which means two mother-child pairs to intervention group per two pairs of control participants. For allocation of the participants an appointment order list for NELLI 5-years follow-up study will be used. Mothers will be randomized to intervention or control group by using sealed envelopes in contact of sampling for NELLI-study.

Power calculations for the study (Table 3.) show that approximately 91 mother and child -pairs are needed, if there are 80 % sedentary women at baseline and the anticipated difference in sedentariness is 25 % between the ACC (accelerometer) and ACC + DVD (accelerometer plus movement-to-music video program) groups after intervention. If the anticipated difference between groups is smaller (than 25 %), the number of pairs needed is 135 per group. In this study sedentary women are defined as being sitting or in reclining posture more than 76 % of waking hours (Husu et al. 2014).

Table 3. Power calculations for primary outcome of the study.

Primary aim: decrease sedentariness of participants by using DVD musical video			
Proportion of sedentary women 80 %	Accelerometer and DVD group (ACC+DVD)	Accelerometer group (ACC)	Number of participants (mother + child pair) needed
α= 0.05, β=0.80, difference between the groups 20 %	60 %	80 %	135/group
difference between the groups 25 %	55 %	80 %	91/group

α= significance level, β=power of the test

Interventions. Mothers and children randomized to accelerometer group (ACC group) will wear to accelerometer belt for first 14 days (two weeks) and last 7 days (during week 8, Figure 4.). Mothers and children randomized to movement-to-music -group (ACC + DVD group) will do the same. Both groups will have questionnaires at baseline, after 2 and 8 weeks and both groups will fill exercise diary during weeks 1, 2 and 8.

In addition movement-to-music -group get either DVD or YouTube-link by e-mail or mobile phone according their own choice at the end of the first week. Mothers in movement-to-music -group will be expected to spend time every second day watching program from DVD player or internet together with their 4–7 years old child. Participants in movement-to-music group will use the BMRI-2 to assess motivational quality of music used in music-to-movement video.

Figure 4. Intervention for 8 weeks.

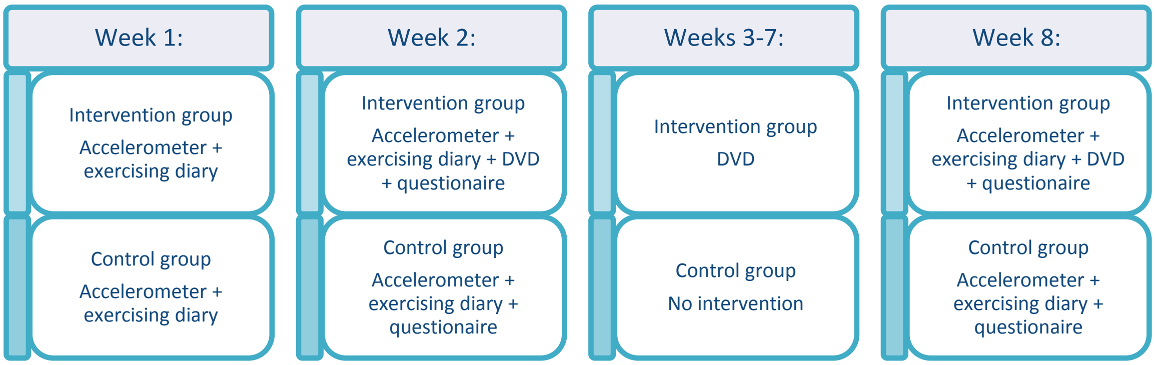

Statistical methods. Effect of the intervention will be analyzed following the intention-to-treat principle by comparing the changes in the main outcomes between intervention and control group participants. Multivariate methods corresponding to analysis of covariance and logistic regression will be used. Non-parametric methods will also be utilized if needed. In the analysis physical activity, sedentary time and body weight of the intervention and control group will be compared. Subgroup analysis in intervention group will be conducted for mother's motivation to exercise with movement-to-music video program and effects of musical background.

4. *Ethical permission and trial registration*

Ethical permission will be requested from Pirkanmaa ethics committee in September 2014. This research project will be accomplished according to good scientific practice, including ethical issues.

Trial registration will be done for ClinicalTrial.gov (National Institutes of Health) in August 2014 before the study begins in September 2014.

5. *Project risks and their management*

The main risks in new data collection for NELLI 5-years follow-up study and Moving Sound -sub-study are related to response rate, which was approximately 50 % in the NELLI one-year follow-up data collection. Therefore, extra efforts will be performed to increase the participation to the 5-year data collection. These efforts include utilization of social media (Facebook closed group), with motivating quizzes, provision of feedback on the results of the earlier studies, and also the possibility to pose questions to the study team. Another effort is related to the possibility of using electronic questionnaires, in case the women are not willing to participate in the tests of NELLI 5-years follow-up study.

Another risk in data collection will be the use of hip-worn accelerometer and its ability to measure movement during movement-to-music video use. There are lots of movements shaking and jiggling hands and/or legs in video program and it is unsure how these kinds of movements can be detected by Hookie-accelerometer. However, Hookie-accelerometer is able to detect overall physical activity and sedentary behavior.

6. *Implementation and funding*

The preliminary study timetable is shown in table 4. Estimated time for starting the Moving Sound intervention is autumn 2014 after the pilot experiences, permission of Pirkanmaa Ethics Committee and trial registration for ClinicalTrial.com (National Institutes of Health).

Table 4. Moving Sound milestones.

	2014 4-5	2014 6-8	2014 9-12	2015	2016	2017
Initial research proposal	■					
Video finishing, BMRI-2 tests and DVD production	■	■				
Pretests of accelerometers, exercising diary and questionnaires	■					
Pilot study	■	■				
Analysis of the pilot study		■	■			
Updating research proposal			■	■		
Writing out the first publication (pilot results, protocol)				■		

The budget of the proposal (Table 5.) includes salaries for one postdoctoral and one researcher, one statistician and other costs. Funding for this trial originates from Academy of Finland research grant and Competitive Research Funding from Pirkanmaa Hospital District for Riitta Luoto and the research group for 4 years' time (2014-2017). The trial is linked to larger cohort study (Pregnancy as a window to future health of mothers and children, NELLI 5-years follow-up). Additional funding for doctoral researcher (Pipsa Tuominen) will be applied from Juho Vainio Foundation, Finnish Cultural Foundation, and Yrjö Jahnsson Foundation during autumn 2014.

Table 5. Personnel and justifications of the funding

	2014, €	2015, €	2016, €	2017, €	Total, €
Postdoctoral researcher	38400	38400	38400	38400	153600
Researcher NN (part-time in 2014)	20000	30000	30000	30000	110000
Statistician	11200	11200	11200	11200	44800
Indirect employee cost (30 %)	20880	23880	23880	23880	92520
Employment cost total, without indirect costs	69600	79600	79600	79600	308400
Other costs:					
Accelerometers (90€/each, 100)	3000	3000	3000	---	9000
Video (2000€) and DVD production (3,50€/each, 350)	2700	525	---	---	3225
Services: language check-ups (1000 €/each)	1000	1000	1000	1000	4000
Travel expenses (Train Helsinki-Tampere 70 €/trip, 5 trips/year)	1050	1050	1050	1050	4200
Congress trips	1000	1000	1000	1000	4000
Total (employee + other costs)	96530	109530	109530	106530	422120
Overheads share (15%)	14480	16430	16430	15980	63318
Total cost of the project	113710	126485	125960	122510	488663

7. Researchers and research environment

The project is multidisciplinary and the collaborating organizations are UKK Institute, Finnish Institute for Occupational Health and Sibelius-Academy. The members of the research teams are shown below (Table 6.). All co-workers will participate in planning the analyses and reporting the results in scientific papers. The UKK Institute for Health Promotion Research, Tampere, owns the data and is responsible for its storage and use. The personal data will be stored for 20 years.

Table 6. Collaborators/researchers and their roles in the study proposal.

Collaborating research organizations and persons	Role/ expertise areas in the study
**UKK Institute for Health Promotion**	
Riitta Luoto, MD, PhD, research director	Principal investigator of the NELLI study
Jani Raitanen, MSc (statistics)	Statistics
Pauliina Husu, PhD, postdoctoral researcher	Physical activity and sedentary behavior, objective measurement
Pipsa Tuominen, MSc (music education), MSc (sports and exercise medicine), researcher	Moving Sound -study, connection between music and physical activity
**Finnish Institute of Occupational Health**	

Minna Huotilainen, PhD, research professor	Cognitive science, consulting role
Laura Sokka, MSc (psychology), researcher	Psychology, consulting role
**Sibelius-Academy, dpt of music education**	
Eeva-Leena Pokela, vice head in Music Education, lecturer	Music Education, Movement-to-music video
Soili Perkiö, lecturer	Music Education, Movement-to-music video

8. Expected research results

The NELLI study was effective in reducing the number of new-born with large birth weight for their gestational age (Luoto et al 2011). Since the NELLI study was one of the first trials aiming to prevent gestational diabetes and having adequate sample size to show effects of the lifestyle intervention cardio-metabolic consequences, also the follow-up results will be important both scientifically and from clinical point of view. The follow-up of mothers and children in the NELLI study will bring unique information concerning children's obesity, which is one of the key public health questions in the future.

Moving sound -intervention will aim to evaluate the practical implementation of physical activity measurement and motivational music programs together. Given the well-known positive effects of music on mood improvement, positive results are expected also with respect to exercise adherence. For example, if children's programs in TV included more musical elements or other features known to decrease sedentariness among children, the results of this intervention study could have larger potentials and effectiveness in Finland.

Additionally, as mentioned earlier the use of music and video together can give an added benefit to increase physical activity for those mothers, who has difficulties to exercise out of home with small children. This study gives a possibility to achieve long term health benefits by moving at home. In this way this study will show one way to prevent and improve the forthcoming health problems.

9. References

Aittasalo M, Pasanen M, Fogelholm M, Kinnunen TI, Ojala K, Luoto R. Physical activity counseling in maternity and child health care - a controlled trial. BMC Women's Health 2008;8:14.

Atkinson G, Wilson D, Eubank M. Effects of music on work-rate distribution during a cycling time trial. International Journal of Sports Medicine 2004;25(8):611–615.

Bassett DR, Rowlands A, Trost SG. Calibration and validation of wearable monitors. Med Sci Sports Exerc 2012;44(S1):S32-S38.

Celis-Morales CA, Perez-Bravo F, Ibanez L, Salas C, Bailey ME, Gill JM. Objective vs. self-reported physical activity and sedentary time: effects of measurement method on relationships with risk biomarkers. PLoS One 2012;7(5):e36345.

Cevasco AM, Kennedy R, Generally NR. Comparison of Movement-to-Music, Rhythm Activities and Competitive Games on Depression, Stress, Anxiety and Anger of Females in Substance Abuse Rehabilitation. Journal of Music Therapy 2005;(42)1, 64–80.

Clark BK, Sugiyama T, Healy GN, Salmon J, Dunstan DW, Owen N. Validity and reliability of measures of television viewing time and other non-occupational sedentary behaviour of adults: a review. Obesity reviews 2009;10:7-16.

Cnattingius S, Villamor E, Lagerros YT, Wikström A-K, Granath F. High birth weight and obesity- a vicious cycle across generations. Int J Obesity 2012;36:1320-24.

Crust L, Clough PJ. The influence of rhythm and personality in the endurance response to motivational asynchronous music. Journal of sports sciences 2006;24(2): 187–195.

Freedson P, Bowles HR, Troiano RP, Haskell W. Assessment of physical activity using wearable monitors: recommendations for monitor calibration and use in the field. Med Sci Sports Exerc 2012;44(1S):S1-S4.

Hagstromer M, Ainsworth BE, Oja P, Sjostrom M. Comparison of a subjective and an objective measure of physical activity in a population sample. J Phys Act Health 2010;7(4):541-50.

Hamburg, J. & Chair, A.A. The Effects of a Movement with Music Program on Measures of Balance and Gait Speed in Healthy Older Adults. Journal of Music Therapy 2003;40(3), 212–226.

Hilakivi-Clarke L, De Assis S, Raitanen J, Luoto R. Pre-pregnancy BMI, gestational weight gain, adiponectin and leptin-results from a clinical trial. Food and Nutrition Sciences, 2012, 3, 556-567 doi:10.4236/fns.2012.34078

Hilakivi-Clarke,L. and de Assis S. Fetal origins of breast cancer, Trends Endocrinol.Metab, 2006 ; Nov;17(9):340-8.

Intille SS, Lester J, Sallis JF, Duncan G. New horizons in sensor development. Med Sci Sports Exerc 2012;44(S1):S24-S31.

Karageorghis CI, Jones L, Low DC. Relationship between exercise heart rate and music tempo preference. Res Q Exerc Sport 2006;77(2):240–250.

Karageorghis CI. The scientific application of music in sport and exercise. In: Sport and Exercise Psychology. Ed: Lane A. London: Hodder Education. 2008; 109-138.

Karageorghis CI, Jones L, Stuart DP. Psychological effects of music tempi during exercise. International Journal of Sports Medicine 2008;29:613-619.

Karageorghis CI, Priest D-L. Music in the exercise domain: a review and synthesis (Part I). International review of Sport and Exercise Psychology 2012;5:44-66.

Karageorghis CI, Priest D-L, Terry PC, Chatzisarantis NLD, Lane AM. Redesign and initial validation of an instrument to assess the motivational qualities of music in exercise: The Brunel Music Rating Inventory-2. Journal of Sports Sciences 2006;24:899-909.

Katzmarzyk PT, Church TS, Craig CL, Bouchard C. Sitting time and mortality from all causes, cardiovascular disease, and cancer. Med Sci Sports Exerc 2009;41(5):998-1005.

Kinnunen TI, Pasanen M, Aittasalo M, Fogelholm M, Hilakivi-Clarke L, Weiderpass E, Luoto R. Preventing excessive weight gain during pregnancy - a controlled trial in primary health care. Eur J Clin Nutr 2007;61:884-91.

Lee IM, Shiroma EJ, Lobelo F, Puska P, Blair SN, Katzmarzyk PT, et al. Effect of physical inactivity on major non-communicable diseases worldwide: an analysis of burden of disease and life expectancy. Lancet 2012;380(9838):219-29.

Luoto R, Kinnunen T, Aittasalo M, Kolu P, Raitanen J, Ojala K, Mansikkamäki K, Lamberg S, Komulainen T, Vasankari T, Tulokas S. Primary prevention of gestational diabetes mellitus and newborn's high birthweight by lifestyle counseling –a cluster-randomized controlled trial. Plos Medicine 2011; http://www.plosmedicine.org/article/info%3Adoi%2F10.1371%2Fjournal.pmed.1001036

Luoto R, Kinnunen TI, Aittasalo M, Ojala K, Mansikkamäki K, Toropainen E, Kolu P, Vasankari T. Prevention of gestational diabetes: design of a cluster-randomised controlled trial and one year-follow-up. BMC Pregnancy and Childbirth 2010;10:39.

Matthews CE, Moore SC, George SM, Sampson J, Bowles HR. Improving self-reports of active and sedentary behaviors in large epidemiologic studies. Exercise and Sport Sciences Reviews 2012;40(3):118-26.

Mathews RM, Clair AA, Kosloski K. Keeping the beat: use of rhythmic music during exercise activities for the elderly with dementia. Am J Alzheimers Dis Other Demen 2001;16(6), 377–380.

Murphy VE, Smith R, Warwick BG, Clifton VL. Endocrine regulation of human fetal growth: the role of the mother, placenta and fetus. Endocrine Rev 2006;27:141-69.

Murrock CJ. The effects of music on the rate of perceived exertion and general mood among coronary artery bypass graft patients enrolled in cardiac rehabilitation phase II. Rehabilitation nursing 2002;27(6), 227–231.

Mustila T, Raitanen J, Keskinen P, Saari A, Luoto R. Lifestyle counseling during pregnancy and offspring weight development until four years of age: follow-up study of a controlled trial. J Negat Results Biomed. 2012 May 8;11:11. doi: 10.1186/1477-5751-11-11.

Patel AV, Bernstein L, Deka A, Feigelson HS, Campbell PT, Gapstur SM, et al. Leisure time spent sitting in relation to total mortality in a prospective cohort of US adults. Am J Epidemiol 2010;172(4):419-29.

van Poppel MN, Chinapaw MJ, Mokkink LB, van Mechelen W, Terwee CB. Physical activity questionnaires for adults: a systematic review of measurement properties. Sports Medicine 2010;40(7):565-600.

Potteiger JA, Schroeder JM, Goff KL. Influence of music on ratings of perceived exertion during 20 minutes of moderate intensity exercise. Perceptual and motor skills 2000;91(3Pt1):848–854.

Sedentary Behavior Research Network. Letter to the Editor: Standardized use of the terms "sedentary" and "sedentary behaviours". Sedentary Behaviour Research Network. Appl. Physiol. Nutr. Metab. 2012;37:540-542.

Siedliecki S, Good M. Effect of music on power, pain, depression and disability. Journal of Advanced Nursing 2006;54(5):553–562.

Terry PC, Karageorghis CI. Psychophysical effects of music in sports and exercise: An update theory, research and application (Abstract). In M. Kattsikis (Ed.), Proceedings of the 2006 Joint Conference of the APS and the NZPS (pp. 415-419). Melbourne, VIC: Australian Psychological Society.

Thorp AA, Owen N, Neuhaus M, Dunstan DW. Sedentary behaviors and subsequent health outcomes in adults a systematic review of longitudinal studies, 1996-2011. American Journal of Preventive Medicine 2011;41(2):207-15.

Vähä-Ypyä H, Vasankari T, Husu P, Suni J, Sievänen H. A universal, accurate intensity-based classification of different physical activities using raw data of accelerometer. Clin Physiol Funct Imaging 2014;Jan 7. doi: 10.1111/cpf.12127. [Epub ahead of print]

Yamasita S, Iwai K, Akimoto T, Sugawara J, Kono I. Effects of music during exercise on RPE, heart rate and the autonomic nervous system. J Sports Med Phys Fitness 2006;46(3):425–430.
